# The Fluoride Ion Affinity Revisited: Do We Need the Anchor‐Point Approach?

**DOI:** 10.1002/chem.202404662

**Published:** 2025-04-22

**Authors:** Morten Lehmann, Salimot Natalie Balogun, Marc Reimann, Martin Kaupp

**Affiliations:** ^1^ Technische Universität Berlin Institut für Chemie 10623 Berlin Germany; ^2^ Universität Innsbruck Institut für Ionenpyhsik und Angewandte Physik 6020 Innsbruck Austria

**Keywords:** Lewis acidity, fluoride ion affinity, anchor-point approach, coupled-cluster calculations, density functional calculations

## Abstract

A large and diverse high‐level benchmark data set of computed gas‐phase fluoride ion affinities (FIAs) for 71 small main‐group Lewis acids is presented. It has been used to evaluate quantitatively DFT approaches with 52 functionals and 4 composite methods. Two widely used indirect anchor‐point methods based on isodesmic reactions with fluorophosgene or the trimethyl silyl cation are compared to the direct computation of the FIA. It has been frequently stated that anchor‐point methods are to be strongly preferred over direct FIA computations at DFT levels, as they avoid treatment of the naked fluoride ion. Here it is shown that this widespread assumption does not hold when modern functionals with low self‐interaction errors and suitable basis sets with diffuse functions are used. In these cases, an anchor‐point approach based on ${\mathrm{OCF}}_{2}$ has little or no advantage, and the widely used anchor‐point calculations based on ${\mathrm{Me}}_{3}{\mathrm{Si}}^{+}$ even deteriorate results in most cases. It is shown that this is due to a break‐down of often prevailing error cancellations in the anchor‐point approach that help to improve results when using less suitable functionals or basis sets. Overall, the direct computation of FIAs at appropriate DFT levels including diffuse basis functions is the clearly preferable route.

## Graphical abstract

A state‐of‐the‐art benchmark of fluoride‐ion affinities has been generated. Its application shows that widely used computational anchor‐point methods based on isodesmic reactions rely on non‐systematic error cancellations and are advantageous only when inappropriate density functionals and/or basis sets without diffuse functions are used. In all other cases direct FIA computations are accurate and preferable.

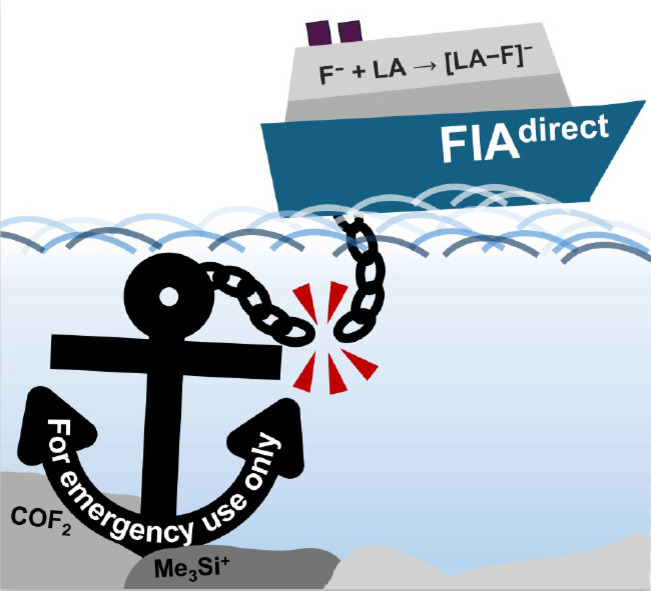

## Introduction

The concept of Lewis acids and bases (or electron pair acceptors and donors) is fundamental in chemistry, and Lewis acid‐base interactions are crucial in a wide range of fields. A Lewis acid can activate a substrate, e. g., as a catalyst,[^1^, ^2^] and its Lewis‐acid strength is an important aspect in fine‐tuning reactivity.^[3]^ Unfortunately, there is no unique way to define the strength of a Lewis acid, albeit base‐independent measures like the global electrophilicity index^[4]^ have been suggested. Unlike Brønsted theory, where acidity is uniquely connected to the heterolytic dissociation of the bond to a proton (albeit the character of this proton does of course change with solvent),^[5]^ the Lewis acidity depends on the base chosen as a reaction partner. One commonly chosen partner is the fluoride ion, and the fluoride ion affinity (FIA) of a Lewis acid is indeed most often taken as a measure of acid strength.^[6]^ Since the fluoride ion is a hard base, this scale can be interpreted as a scale of the strength of a Lewis acid against hard Lewis bases. For estimating the interaction with softer Lewis bases the FIA might be less well suited. Nevertheless, it is solidly established in the field. FIAs are most often estimated using quantum‐chemical methods, and the best approach to do such calculations is the main focus of this work.

**Figure 1:**
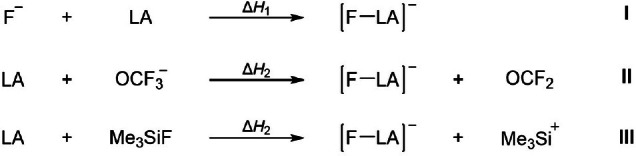
Reaction scheme for the reaction defining the FIA directly (I) and for the (pseudo)isodesmic reactions with ${\mathrm{OCF}}_{2}$ (II) and ${\mathrm{Me}}_{3}{\mathrm{Si}}^{+}$ (III) defining the anchor‐point approach.

The FIA is defined and can be directly calculated as the negative of the enthalpy of the reaction of a Lewis acid with a fluoride ion in the gas phase (see reaction I in Figure 1):[^6^, ^7^, ^8^]

(1) $${\mathrm{FIA}}^{\mathrm{direct}}=-\Delta H_{1}$$

The experimental determination of FIAs is non‐trivial, and the accuracy of the results is often low. Depending on the actual system and the applied method, the uncertainty is often significantly more than ±2 kcal/mol (>8 kJ/mol),[^5^, ^8^, ^9^, ^10^] as gas‐phase experiments are generally demanding, while solvent effects may in any case change the picture in solution. For the systematic comparison of Lewis‐acid strength one typically relies on the quantum‐chemical screening of gas‐phase FIAs. Interestingly, this is rarely done directly using Eq. (1). Instead one usually employs the so‐called anchor‐point method, where a (pseudo)isodesmic reaction with a reference compound, LA* such as ${\mathrm{OCF}}_{2}$ , is used (see reaction II in Figure 1).[^6^, ^7^] The FIA of this reference is assumed to be known accurately, either from experiment or from very high‐level computations. Then the FIA of the compound in question becomes:
(2)

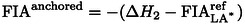




Other authors^[11]^ argued for the use of ${\mathrm{Me}}_{3}{\mathrm{Si}}^{+}$ as the reference Lewis acid (see reaction III in Figure 1). In principle, this could have the advantage of forming a neutral rather than anionic complex,^[12]^ and it links the method to similar treatments for other anion affinities, e. g., for hydride or chloride.^[11]^ Any Lewis acid with a well‐established FIA could of course be used to formulate an isodesmic reaction as a basis for an anchor‐point approach.

The usual argument made for using the anchor‐point method rather than direct computations are difficulties in the accurate treatment of free fluoride,[^6^, ^7^, ^11^, ^12^] which are avoided when using the isodesmic reaction. While such difficulties were clearly apparent in earlier works, when computational facilities were more limited,[^6^, ^7^] today the reasons for the cited difficulties in direct computations appear less justified and deserve closer scrutiny. A recent work^[12]^ pointed out that systematic and broad benchmark studies on FIA calculations were missing, as the few previous investigations[^11^, ^13^, ^14^, ^15^] lacked breadth in molecules and/or in the methods evaluated. Ref. [12] therefore provided a benchmark set based on CCSD(T) calculations with extrapolation to the complete basis‐set limit. However, the data still only covers 15 Lewis acids, and only 9 DFT functionals had been evaluated against these data. It was concluded that the direct approach should not be used, and the anchor‐point method based on ${\mathrm{Me}}_{3}{\mathrm{Si}}^{+}$ ^[11]^ was advocated. The suggested functionals and methods have very recently been used to generate DFT data to train machine‐learning models,^[16]^ and to evaluate solvation effects on FIAs.^[17]^

Here we provide a significantly broader state‐of‐the‐art benchmark of gas‐phase FIAs for 71 main‐group Lewis acids and use it to evaluate systematically a much wider range of modern DFT approaches. We arrive at different findings than the abovementioned prior studies, in particular regarding the necessity to use the anchor‐point approach. Based on our analyses, we can dispel some misconceptions and will make recommendations for efficient computational protocols that provide chemical accuracy or near chemical accuracy in computing FIAs for many systems.

## Results and Discussion

### Quality of the Benchmark Data

The FIA71 benchmark data provided here (see Table S1 in Supporting Information) represent direct computations without any anchor point at the CCSD(T*)‐F12a/auc‐cc‐pVTZ‐F12 level (see Computational Details) and are expected to be accurate in almost all cases to within chemical accuracy (i. e. to 1 kcal/mol or ca. 4 kJ/mol). We have estimated remaining basis‐set errors for ${\mathrm{OCF}}_{2}$ and ${\mathrm{SiMe}}_{3}^{+}$ by performing additional CCSD(T*)‐F12a single point calculations using aug‐cc‐pVQZ‐F12 basis sets (see Table S2 in Supporting Information). For both of these anchor‐point systems, changes were well below 1 kJ/mol. Similarly small effects are found for these two cases when going from perturbational to full triple substitutions, i. e. by comparing LNO‐CCSD(T)/aug‐cc‐pVDZ to LNO‐CCSDT/aug‐cc‐pVDZ results (Table S2). When evaluating T_1_ ^[18]^ and D_1_ ^[19]^ diagnostics for the benchmark set, BeO and $F_{3}^{-}$ (the fluoride adduct of F_2_) stand out by far as the systems with the largest multi‐reference character. Indeed, the effect of full triple excitations on the FIA is larger (2 kJ/mol for BeO, 3 kJ/mol for $F_{3}^{-}$ ) for both species. For BeO this is partly compensated by a 1 kJ/mol change from the larger basis sets. This is not the case for $F_{3}^{-}$ (see Table S2). However, in that case adding full quadruple excitations (which have a negligible effect for BeO) provides a compensating correction of opposite sign. Overall, even these two systems are therefore expected to be covered with chemical accuracy by the benchmark. The signficant effect of full quadruple excitations for $F_{3}^{-}$ is consistent with its multi‐reference character noted earlier.^[20]^

### Benchmarking Different XC Functionals, Comparison of Direct and Anchor‐Point Approaches

We use the FIA71 data to evaluate different DFT approaches using direct calculations as well as anchor‐point computations using either ${\mathrm{OCF}}_{2}$ or ${\mathrm{Me}}_{3}{\mathrm{Si}}^{+}$ as reference compound. Note that these computations use large def2‐QZVPD basis sets including diffuse functions to effectively eliminate basis‐set errors. We will come back to basis‐set effects further below. We also note that here we also use the computed benchmark data to establish the anchor‐point FIAs for both ${\mathrm{OCF}}_{2}$ and ${\mathrm{Me}}_{3}{\mathrm{Si}}^{+}$ , i. e. no experimental data enter the picture (see Computational Details).

The FIA (reaction I in Figure 1) consists of the electronic energy difference and a temperature‐dependent enthalpic contribution obtained from the vibrational analysis. As the first part is dominant, and we find the second part to agree closely between our CCSD(T) and various DFT approaches, we only discuss the performance of different XC functionals for the electronic part ($\Delta E^{\mathrm{FIA}}$ ). The relative performance of different functionals does not change when varying the enthalpy contributions. The effect of using structures optimized at different DFT levels is also essentially negligible (see Tables S3–S8 and Figures S1–S3 in Supporting Information). For example, reoptimization of the structures at a given DFT level results in changes below 1 kJ/mol for most functionals (up to 2.8 kJ/mol for the B97‐3c composite method; Figure S1, Table S3). Using different input structures changes direct FIA results for LH20t‐D4 by at most 1.7 kJ/mol (Figures S2–S3, Tables S4, S5). We note in passing that even structures optimized at one of the cheaper “3c” composite methods are well suited to obtain accurate FIAs, when using a better method for the energy calculation.

**Figure 2:**
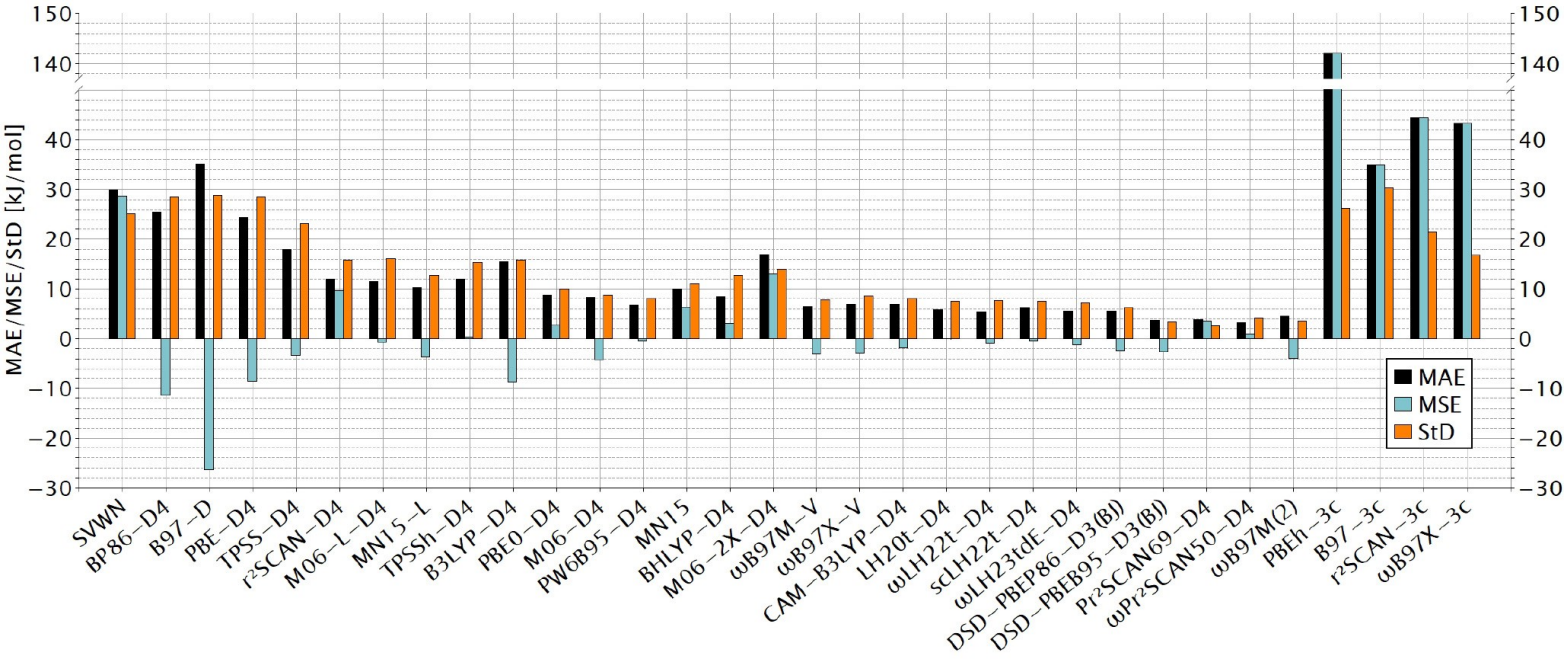
Mean absolute errors, mean signed errors and standard deviations of $\Delta E^{\mathrm{FIA}}$ against the CCSD(T*)‐F12a reference values for a selection of the evaluated XC functionals (with def2‐QZVPD basis sets) and the “3c” composite methods.

Table S9 in Supporting Information reports the final directly computed calculated FIAs for all DFT functionals evaluated, and Table S10 the resulting statistical evaluation. Figure 2 plots the mean absolute error (MAE), the mean signed error (MSE), and the standard deviation (StD) compared to the benchmark data, of the direct DFT computation for a selection of the evaluated XC functionals.

Postponing discussion of the “3c” composite approaches, the local‐density‐approximation (LDA) functional SVWN and the generalized‐gradient‐approximation (GGA) functionals give the largest deviations, with MAEs around 30 kJ/mol. meta‐GGAs improve the results to MAEs somewhat above 10 kJ/mol. Several global hybrids, range‐separated hybrids, local hybrids, and range‐separated local hybrids achieve single‐digit MAEs. Among these rung 4 functionals, the range‐separated local hybrids ω LH22t‐D4 and ω LH23tdE‐D4 perform best (MAE 5.3, 5.4 kJ/mol, respectively), closely followed by the local hybrid LH20t‐D4 (MAE 5.8 kJ/mol). Consistent with their higher rung 5 and their known top performance for main‐group thermochemistry, several double hybrids are the overall best‐performing functionals: in particular Pr^2^ SCAN 69‐D4, DSD‐PBEB95‐D3(BJ), and $\omega {\mathrm{Pr}}^{2}\mathrm{SCAN}$ 50‐D4 achieve MAEs of 3.7 kJ/mol, 3.6 kJ/mol, and 3.2 kJ/mol, respectively. The overall albeit not uniform improvement with the rung of the ladder parallels observations for other main‐group energetics benchmarks, e. g., for the GMTKN55 database.^[21]^ The lowest MAEs are within chemical accuracy, and we should also keep in mind that this may already be in the accuracy range of the benchmark data, and clearly below the usually much larger errors of experimental FIAs. The composite methods of the “3c” family exhibit much larger errors (MAEs between 142.0 kJ/mol for PBEh‐3c and 34.9 kJ/mol for B97‐3c), likely reflecting errors due to their inherently smaller “built‐in” basis sets (see below) that are not compensated sufficiently by the included correction terms. This is accompanied by large positive MSEs.

Coming back to the regular XC functionals, Figure 1 shows that for most of them the MSE is negative, indicating a systematic underestimate of the FIA (SVWN, $r^{2}\mathrm{SCAN}$ , MN15, BHLYP‐D4, and M06‐2X‐D4 are notable exceptions). Several of the best‐performing functionals have very small negative MSEs, indicating a largely statistical distribution of the deviations. The standard deviations are also largest for the rung 1 and 2 functionals and smallest for the best‐performing rung 5 functionals. Overall we can conclude that a significant number of XC functionals are clearly suitable for the “direct” computation of FIAs without use of an anchor‐point approach, as indicated by three functionals with MAEs within chemical accuracy and eleven more functionals with MAEs below 8 kJ/mol.

**Figure 3:**
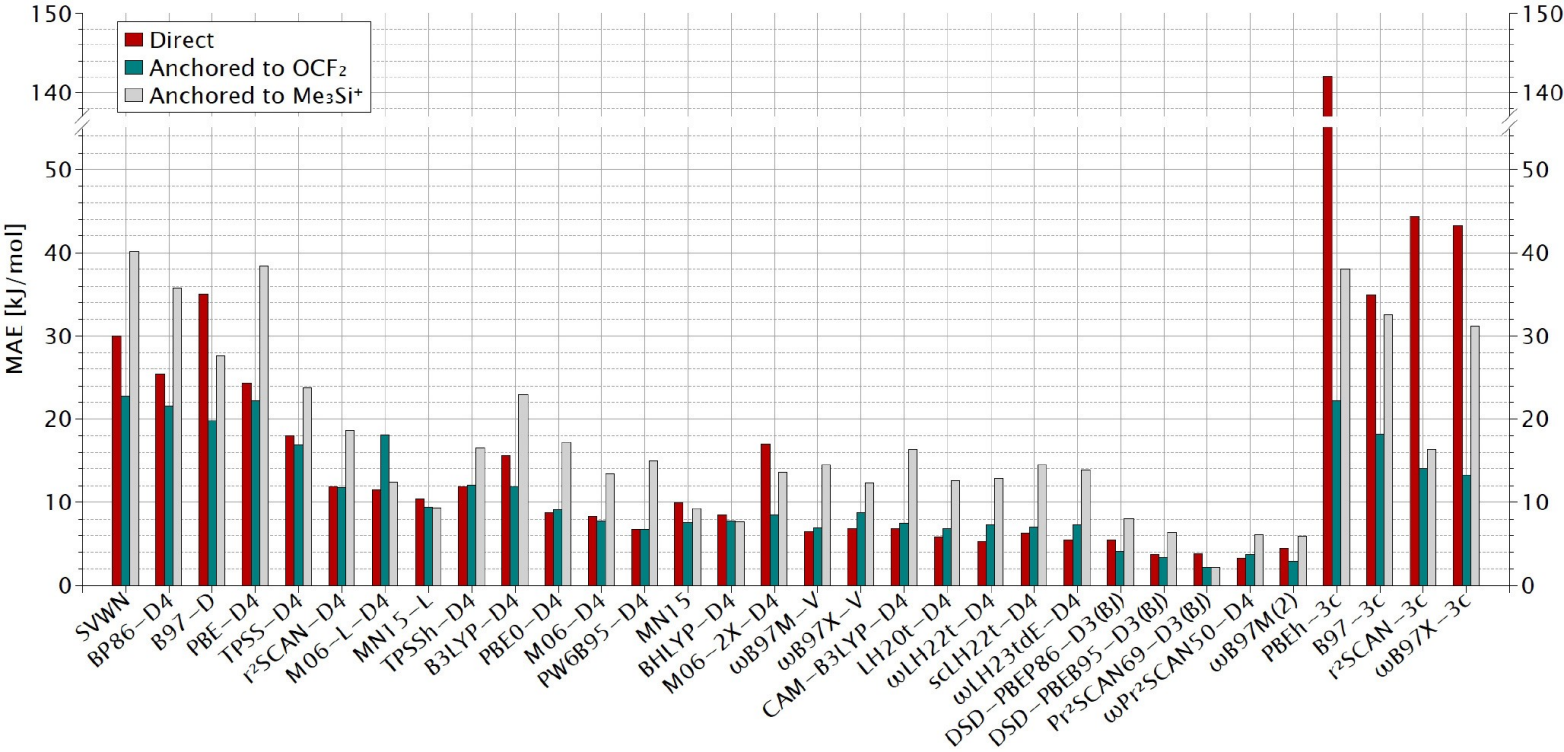
MAE against the CCSD(T*)‐F12a reference values for a selection of the evaluated XC functionals and “3c” composite methods obtained with the three different approaches.

We have subsequently computed the same $\Delta E^{\mathrm{FIA}}$ values using the anchor‐point approach (2) with either ${\mathrm{OCF}}_{2}$ or ${\mathrm{Me}}_{3}{\mathrm{Si}}^{+}$ as reference compound, using again def2‐QZVPD basis sets. This allows us to compare the MAEs for the direct and the two anchor‐point approaches in Figure 3 (cf. Table S11 in Supporting Information for full statistical data). For those functionals that exhibited larger deviations in the direct calculations, the ${\mathrm{OCF}}_{2}$ ‐based computations indeed improve performance. This holds for the rung 1 and rung 2 functionals, for a few global hybrids (B3LYP‐D4, M06‐2X‐D4), and for the four composite schemes. However, the situation is different for the better‐performing functionals, where no significant improvement is seen, and the ${\mathrm{OCF}}_{2}$ ‐based MAEs are indeed often larger than the direct‐computation results. The latter observation holds for several range‐separated hybrids, local hybrids, and range‐separated local hybrids. In case of the best‐performing double hybrids, differences between the direct and ${\mathrm{OCF}}_{2}$ ‐based MAEs may in fact be below the estimated accuracy of the reference data and therefore insignificant. That is, a significant improvement with the ${\mathrm{OCF}}_{2}$ ‐based anchor‐point method is only seen for inferior functionals or composite methods.

Turning to the ${\mathrm{Me}}_{3}{\mathrm{Si}}^{+}$ ‐based anchor system, we obtain an initially rather surprising picture: except for the four composite methods, as well as for B97‐D, MN15‐L, MN15, BHLYP‐D4, and one double hybrid, the deviations actually become larger than for the direct computations, in many cases significantly so! This holds even for most rung 1 and rung 2 functionals, and in most cases the ${\mathrm{Me}}_{3}{\mathrm{Si}}^{+}$ ‐based data are also worse than the ${\mathrm{OCF}}_{2}$ ‐based ones. These data therefore provide very little justification for using the ${\mathrm{Me}}_{3}{\mathrm{Si}}^{+}$ ‐based anchor‐point approach at all (but see below).

### The Importance of the Basis Set

**Figure 4:**
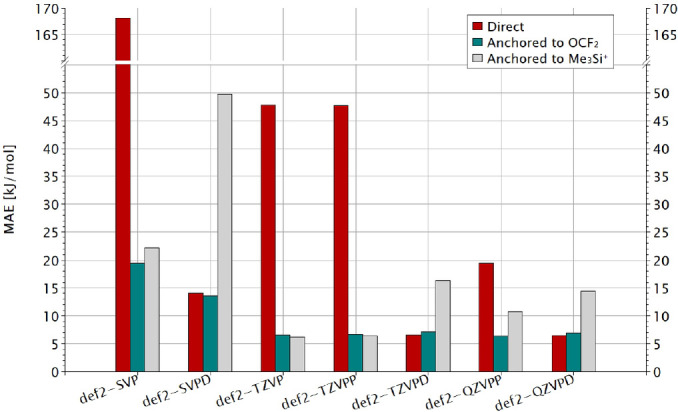
MAE against the CCSD(T*)‐F12a reference values obtained for the three different approaches for different basis sets with ω B97M‐V.

The larger deviations of the composite “3c” approaches hint already at the importance of basis‐set effects (the errors are largest for PBEh‐3c, which utilizes small def2‐mSVP basis sets). In these cases improvement of the FIAs by using the ${\mathrm{OCF}}_{2}$ ‐based anchor‐point method, and the smaller improvements by the ${\mathrm{Me}}_{3}{\mathrm{Si}}^{+}$ ‐based ones, reflects a compensation of basis‐set errors. As we had used large, likely almost converged def2‐QZVPD basis sets for the regular DFT calculations, it becomes important to get insight into the basis‐set requirements. We have therefore evaluated different basis sets from the “def2 family” for one of the better‐performing rung 4 functionals, ω B97M‐V, comparing again the direct and the two anchor‐point approaches. Full numerical data and statistics can be found in Tables S12 and S13, respectively, in Supporting Information. Figure 4 plots the resulting MAEs.

As expected, the largest MAEs of the direct calculation are found for the smaller basis sets, improving along the series def2‐SVP, def2‐TZVP, def2‐TZVPP, and def2‐QZVPP. In those cases the anchor‐point methods provide a clear improvement, consistent with prior conventional wisdom that DFT calculations of FIAs should be performed within an anchor‐point scheme. Note, however, that adding only one set of diffuse functions to the smallest def2‐SVP basis set (def2‐SVPD) almost eliminates the advantage of the ${\mathrm{OCF}}_{2}$ ‐based over the direct approach, while the ${\mathrm{Me}}_{3}{\mathrm{Si}}^{+}$ ‐based data are now clearly inferior. Note in particular that in the latter case the MAE *with* diffuse functions is now larger than without! This holds true for all basis sets that include diffuse functions, albeit not always to such an extent. As soon as we include diffuse functions, the direct FIA computations become competitive with the given ω B97M‐V functional, and there is no good reason to use an anchor‐point method. We also note that large def2‐QZVP or def2‐QZVPP basis sets still produce larger MAEs in the direct calculations than the much smaller def2‐SVPD basis (Table S12), emphasizing the crucial importance of diffuse functions even further, while less diffuse polarization functions are much less effective. In fact, the decisive role of the diffuse functions is in the description of the free fluoride ion. Just adding a diffuse set to all fluorine atoms therefore provides most, albeit not all, of the necessary improvement (Table S13). A def2‐TZVPD basis does already provide results that are very close to those with the largest def2‐QZVPD basis set, suggesting possible significant computational savings for larger systems while obtaining still very accurate data, with an almost converged basis set.

Looking only at the individual MAEs in Figure 4, one could say that anchor‐point calculations with “non‐diffuse” basis sets like def2‐TZVP or def2‐TZVPP perform competitively with the direct calculations when diffuse def2‐TZVPD or def2‐QZVPD basis sets are used. That is, the resulting data still convey a reasonable picture of FIAs. But due to the abovementioned non‐systematic error compensation involved, at least the data obtained with the ${\mathrm{Me}}_{3}{\mathrm{Si}}^{+}$ anchor point should be treated with great care, given that the data deteriorate even from def2‐TZVPP to def2‐QZVPP and even more so when adding explicitely diffuse functions.

As one argument for sticking with anchor‐point approaches could be the increased computational cost of adding diffuse functions, we have chosen the FIA of the common, somewhat larger Lewis‐acid $B{\left(C_{6}F_{5}\right)}_{3}$ to compare basis‐set effects on wall times of the computations (again for the range‐separated hybrid ω B97M‐V; Tables S14 and S15). Benchmarking of ω B97M‐V FIAs is made against local‐correlation PNO‐CCSD(T*)‐F12a data, which in turn agree excellently with our canonical coupled‐cluster reference data for smaller systems (Table S14). Adding a set of diffuse functions to def2‐TZVP to give def2‐TZVPD on all atoms increases the wall time by about 75 % when using analytical four‐center exchange integrals and only by 33 % when using semi‐numerical integration for exact exchange and RI‐J for the Coulomb integrals (Table S15). Including diffuse functions only on all fluorine atoms gives virtually identical FIA results with an even smaller increase in computational cost of 25 % and 11 %, respectively, in spite of the relatively large number of fluorine atoms in the system. As the negative charge in this larger anion is rather delocalized, it is in fact sufficient to add diffuse functions only to the free and bound fluoride ion, at negligible cost. Consistent with the above results for the wider test set, the ${\mathrm{SiMe}}_{3}^{+}$ ‐based anchor‐point method again gives larger MAEs when diffuse basis functions are included (Table S15), again clearly demonstrating non‐systematic behavior. Given these findings, there is really no good reason nowadays in molecular calculations to prefer anchor‐point methods. As a possible exception, the use of such isodesmic equations might still be justified for densely packed periodic solids with large unit cells, where computations with hybrid functionals and with diffuse basis sets could be limited.

The importance of diffuse functions in the description of anions has of course been known for a long time in other contexts.[^22^, ^23^, ^24^] The prior conventional wisdom suggesting the preferable use of anchor‐point approaches in computing FIAs therefore reflects not only the earlier unavailability of more accurate XC functionals that have been developed over the past decades, but also insufficiently large basis sets used in most earlier works. For the ${\mathrm{Me}}_{3}{\mathrm{Si}}^{+}$ ‐based anchor approach we see most clearly that the error compensation obtained for small basis sets without diffuse functions fails significantly when adequate basis sets with diffuse functions are used. This causes an unpredictable basis‐set dependence of the MAE for the anchor‐point method. In contrast, the direct approach shows a systematic and logical trend, which is certainly preferable.

Additional test evaluations of the basis‐set convergence for selected double hybrids (Table S16 in Supporting Information) indicate the basis‐set errors in the presence of diffuse functions to be within the accuracy of the reference data even in this case. While def2‐QZVPD basis‐set results still deviate by up to 6 kJ/mol for the EA of the fluorine atom from the CBS(Q,5,6) limit in selected double hybrid calculations, deviations for selected FIAs remain below 3 kJ/mol even for the most sensitive Lewis acids like ${\mathrm{ClF}}_{5}$ (Table S16). We may thus transfer the above conclusions also to double hybrids, even though one might expect somewhat larger basis‐set dependencies due to the presence of perturbational contributions to the correlation energy.

### Reevaluation of Literature Statements

In light of the presented data, we may now reevaluate a number of statements made in the past on the computation of FIAs. We should start by noting that anchor‐point approaches work by shifting the computed FIAs for a given method by a constant amount. The relative order of different Lewis acids is not affected. We can show this by taking the difference between direct and anchored FIA for a given method:
(3)

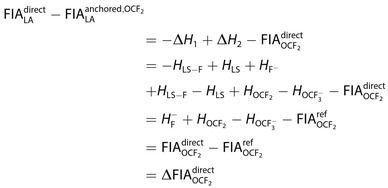




Assuming that ${\mathrm{FIA}}_{{\mathrm{OCF}}_{2}}^{\mathrm{ref}}$ is accurate, we see that the constant shift simply reflects errors of the given method for the computation of ${\mathrm{OCF}}_{2}$ , ${\mathrm{OCF}}_{3}^{-}$ , and $F^{-}$ . The first two numbers are specific for a given reference compound, whereas all anchor‐point approaches attempt to cancel out errors made in the treatment of the free fluoride ion. Given the constant shift, we can already clearly refute statements that the direct approach might behave “erratically” compared to the anchor‐point method,^[15]^ eliminating one argument in favor of the latter.

**Figure 5:**
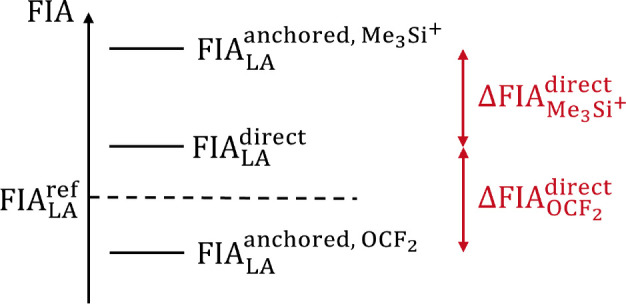
Illustration of the relations between the directly calculated and anchor‐point values for a given Lewis acid LA. Δ indicates the deviation from the exact result.

Eq. (3) shows that the constant shift can be interpreted as the error made for the directly calculated FIA of the anchor system. Assuming that this error is similar to that for any other FIA studied, subtracting it will improve the result. It is trivial, but still important to underline, that a failure in this error compensation will deteriorate the results. Essentially one eliminates the errors made in computing $F^{-}$ , and one hopes that the errors made for ${\mathrm{OCF}}_{2}$ and ${\mathrm{OCF}}_{3}^{-}$ cancel in part with the errors made for the given Lewis acid and its fluoride adduct. Similar arguments hold for any other anchor system. We also note the following: if the error in the directly computed FIA for ${\mathrm{OCF}}_{2}$ were entirely due to the treatment of free fluoride, the anchor‐point method should give exact FIAs for any Lewis acid. However, as we have seen above, this is clearly not the case. Figure 5 shows that for a given Lewis acid, switching from a direct to an anchor‐point method may shift the value closer to or farther away from the accurate result.

Based on this understanding of the error cancellations involved, we can interpret the present results in relation to, e. g., the findings of Ref. [12]. Here we find that the ${\mathrm{OCF}}_{2}$ ‐based anchor‐point approach can lead to improvement, deterioration or negligible effects, depending of the method evaluated. Starting with computations using adequate basis sets including diffuse functions, notable improvement is found only for poorly performing functionals. For almost all functionals, the ${\mathrm{Me}}_{3}{\mathrm{Si}}^{+}$ ‐based anchor‐point approach then also produces larger deviations than the ${\mathrm{OCF}}_{2}$ ‐based one. This is in clear contrast to the findings of Ref. [12], where the authors observed a large improvement by the ${\mathrm{Me}}_{3}{\mathrm{Si}}^{+}$ ‐based method over the direct calculation for the majority of functionals studied. Based on our basis‐set evaluations (e. g., Figure 4, Table S13), we can now clearly state that this is due to the lack of diffuse functions in the basis sets used in Ref. [12] (def2‐SVP, def2‐TZVPP, def2‐QZVPP). In the absence of diffuse functions, the dominant errors arise for the free fluoride anion. In contrast, when using adequate basis sets with diffuse functions, the remaining errors are dominated by the given Lewis acid and its fluoride adduct. These errors can vary widely, in particular if we study a wide range of chemically different compounds. Then use of an anchor‐point approach does not necessarily improve results, or only in an accidental way.

It has also been argued[^11^, ^12^] that ${\mathrm{Me}}_{3}{\mathrm{Si}}^{+}$ is a better reference compound than ${\mathrm{OCF}}_{2}$ , due to its cationic nature, which supposedly reduces dynamical correlation errors. This implies that an anchor system with a small error in its directly computed FIA is prefered. This disregards the mechanism of error compensation described above. The error for the anchor system should not be minimal but similar to that for the Lewis acid of interest. This error compensation depends of course on the method used. As we have seen above, when using adequate basis sets, for most functionals the ${\mathrm{OCF}}_{2}$ reference tends to produce smaller deviations (with the notable exception of M06‐L‐D4; Table S11). Again, the previous study did not include diffuse functions, which explains in part the different findings (in any case the better performance with ${\mathrm{Me}}_{3}{\mathrm{Si}}^{+}$ held only for six out of nine functionals studied).^[12]^ We note in passing that the CCSD(T)/CBS(TZ/QZ) reference data used in that work still contain small residual basis‐set incompleteness errors. For example, our reference value for ${\mathrm{BCl}}_{3}$ is 6 kJ/mol lower than that in Ref. [12], and we find that our F12‐based benchmark computations agree better with three‐point basis‐set extrapolation schemes (using triple‐, quadruple‐ and quintuple‐ζ basis sets) than the two‐point TZ/QZ scheme used in Ref. [12] (cf. Table S17 in Supporting Information).

The difficulty in treating computationally the free fluoride ion has often been linked to difficulties in computing the electron affinity (EA) of the fluorine atom.[^6^, ^12^, ^25^] To this end Ref. [12] provided computed fluorine‐atom EAs at different levels. However, also in this case one should use adequate basis sets including diffuse functions. Results with def2‐QZVPD basis sets and various functionals in a “Δ SCF approach” are given in Table S18 in Supporting Information. While the errors obviously depend on the functional, the better‐performing ones achieve values to within 5 kJ/mol from the “experimental” reference value of 330.8 kJ/mol (back‐corrected from experiment to remove relativistic effects for better comparability with the present non‐relativistic computations). Table 1 shows selected results for a few of the top XC functionals for FIAs, comparing def2‐QZVPD results to results with the def2‐QZVPP basis without diffuse functions used in Ref. [12]. The significant impact of diffuse functions is again obvious, as is the very good performance of several functionals when using the larger basis.

**Table 1:** EA (in kJ/mol) of the fluorine atom for different combinations of XC functional and basis set.

	PW6B95	ω B97X‐V	LH20t	ω LH22t	exp.
def2‐QZVPP	301.2	300.7	304.4	308.6	330.8^[a]^
def2‐QZVPD	326.1	325.3	326.9	332.4	

	B2PLYP	DSD‐BLYP	DSD‐PBEP86	DSD‐PBEB95	
def2‐QZVPP	306.3	304.5	299.1	291.8	
def2‐QZVPD	332.9	329.7	323.5	315.8	

[a] As all calculations were performed using non‐relativistic Hamiltonians, it is appropriate to back‐correct the experimental value of 328.2^[12]^ by an estimate for scalar and spin‐orbit relativistic effects (taken here from (X2C)‐MRCI+Q/CBS(T,Q,5) calculations using the 2p orbitals as active orbitals).

As an aside we remark on a related issue, the ongoing discussion regarding the often positive HOMO energies of (atomic) anions like fluoride when using semi‐local functionals.[^24^, ^26^, ^27^, ^28^, ^29^] Without entering that discussion in detail we note that all rung 1–3 functionals in this work indeed give a positive HOMO energy for fluoride. While this is true for B97‐D, which gives the largest deviations for the FIAs in the present work, it holds also for other functionals like, e. g., $r^{2}\mathrm{SCAN}$ , which provides an MAE of 11.9 kJ/mol, comparable to those for a number of rung 4 functionals with negative HOMO energies for fluoride (e. g., B3LYP, MAE 15.5 kJ/mol). This shows that, while positive HOMO energies of fluoride are a manifestation of self‐interaction errors (resulting in the wrong behavior of the DFT potential), these do not automatically invalidate other aspects like FIAs. Notably, while a positive HOMO energy reflects an error in the asymptotic potential, it is not necessarily accompanied by a large density error.^[29]^ As a result, evaluating electron affinities as the energy difference between the neutral and the anionic species (Δ SCF) can still give very good agreement with the experiment (see above), even when connecting the EA to the HOMO energy of the anion via Koopmans’ theorem gives large deviations. This is shown in Table S18 and Figure S4 in Supporting Information for all functionals of this study. Note that most better‐performing functionals of our benchmark study have reduced self‐interaction errors and therefore also negative HOMO energies for the fluoride ion. Some double hybrids with particularly large global or long‐range EXX admixture give the best HOMO energies for fluoride, and several range‐separated hybrids and range‐separated local hybrids also come close. Overall, this means that there is no special problem whatsoever in treating the naked fluoride ion with modern DFT methods, provided that basis sets with diffuse functions are used!

## Conclusions

We have presented the FIA71 benchmark of gas‐phase fluoride ion affinities based on highly accurate, explicitly correlated CCSD(T*)‐F12 calculations and used it to thoroughly evaluate approximate DFT methods from all rungs of the “Jacob's ladder” hierarchy for a wide variety of different main‐group Lewis acids. This has allowed us to revisit and address a number of assumptions made in previous works for such DFT calculations of FIAs.

A main observation is, that direct DFT calculations of FIAs without any anchor‐point approach can provide excellent results within or close to chemical accuracy when a) modern functionals from the higher rungs of the ladder and b) adequate one‐particle basis sets with diffuse functions are used. In fact, as anchor‐point methods, e. g., based on ${\mathrm{OCF}}_{2}$ or ${\mathrm{Me}}_{3}{\mathrm{Si}}^{+}$ reference compounds rely strongly on sometimes non‐systematic error compensation, their use with good functionals and basis sets can be disadvantageous, in particular with ${\mathrm{Me}}_{3}{\mathrm{Si}}^{+}$ . Even a triple‐zeta basis with diffuse functions like def2‐TZVPD is sufficient for accurate direct FIA calculations, and one may even opt to add the diffuse functions only to the fluorine atoms and for larger systems maybe even only to the free and bound fluoride anion. This means that the added computational burden of adding diffuse functions ranges from moderate to negligible. Energy computations with more advanced functionals like local hybrid, range‐separated hybrid, range‐separated local hybrid, or double hybrid functionals also do not incur a particularly large computational burden even for larger molecules, as structure optimizations and frequency calculations can be safely performed at less computationally demanding levels of theory. For most conceivable situations there remains thus no good reason for using isodesmic anchor‐point approaches.

Several double hybrids provide directly computed FIAs to within chemical accuracy (i. e., with MAEs below 4 kJ/mol) or slightly above, followed closely by the range‐separated local hybrids ω LH22t‐D4 and ω LH23tdE‐D4, and the simpler LH20t‐D4 local hybrid (all with MAEs below 6 kJ/mol), and other rung 4 functionals. We note in passing that ω LH23tdE‐D4 includes correction terms for strong correlations and for delocalization errors that provide advantages for settings with appreciable static correlation or with spin‐contamination in open‐shell transition‐metal systems.[^30^, ^31^] This could become useful when applying such functionals to the FIAs of transition‐metal complexes, which have not been included in FIA71.

The reason why previous studies strongly discouraged direct FIA computations with DFT methods and advocated for anchor‐point methods is the use of inadequate basis sets lacking diffuse functions and/or of functionals with larger self‐interaction errors. The basis‐set aspect is particularly crucial. While the FIA71 benchmark is based on high‐level CCSD(T) structures, we found that DFT‐optimized structures perform also well, and DFT calculations are of course more convenient for providing the enthalpic contributions to the FIA. While the present work has focused on gas‐phase FIAs, the transfer of the direct computations to solvent environments is expected to also produce reliable results, as long as possible charge‐assisted hydrogen bonds to the small fluoride ion^[32]^ are adequately accounted for.

## Computational Details

The new high‐level FIA71 benchmark set (cf. Table S1 in Supporting Information) consists of 71 main‐group Lewis acids with up to 6 non‐hydrogen atoms and central atoms from groups 13–17 and from periods 2–6. The computed FIAs range from 45.1 kJ/mol (CO) to 1243.2 kJ/mol ($F_{3}{\mathrm{Si}}^{+}$ ) and thereby cover a vast space of Lewis‐acid strengths. Structures, energies, and harmonic vibrational frequencies of the Lewis acids and their fluoride‐ion adducts were computed using the MOLPRO program package, version 2023.2,[^33^, ^34^] at the coupled‐cluster level including single, double and perturbative triple excitations, CCSD(T), using RHF reference functions. To approach the basis‐set limit, explicitly correlated calculations were performed using the F12a approximation.[^35^, ^36^] Explicit correlation effects on the perturbative triples were estimated by scaling the (T) contribution by the ratio of MP2‐F12 and MP2 correlation energies, denoted as (T*).^[36]^ All calculations employed aug‐cc‐pVTZ‐F12 basis sets for atoms up to Cl and cc‐pVTZ‐PP‐F12 basis sets with the appropriate scalar relativistic effective‐core potentials (ECPs) beyond Ar (denoted AVTZ‐F12 overall in combined form throughout this work) as well as the respective auxiliary basis sets automatically assigned by the MOLPRO program. As the heavier atoms generally function as central atoms in the complexes and do not carry significant negative charge, no additional augmentation functions were added to the cc‐pVTZ‐PP‐F12 valence basis. All calculations employed the frozen core option chosen by MOLPRO as a default. For all atoms beyond Ar, we additionally correlated the (n‐1)d orbitals, using the appropriate pair‐specific geminal exponents of the F12 approach.^[37]^ For certain cases, care must be taken with the frozen‐core approximation at MP2 and CC levels, as the fluorine 2s orbitals may be energetically below the (n‐1)d orbitals, and orbital rotations must be performed to allow for a consistent description. In ${\mathrm{InCl}}_{3}$ , the 4d orbitals of In and the 3s orbitals of Cl are of similar energies and mix in the RHF wave function. Calculations on ${\mathrm{InCl}}_{3}$ and ${\mathrm{InCl}}_{3}F^{-}$ were therefore performed using a single geminal exponent of 1.0 in the F12 ansatz. Structures were optimized up to energy changes below 10^−7^ Hartree and gradient norms below 5 ⋅ 10^−5^ Hartree/bohr. All optimizations were performed in Z‐matrix mode and employed the highest possible symmetries. All structures were verified to be minima by harmonic vibrational frequency analysis. Due to the computational demand of the full calculation of ${\mathrm{IF}}_{6}^{-}$ , vibrational frequency calculations of ${\mathrm{IF}}_{5}$ and ${\mathrm{IF}}_{6}^{-}$ employed aug‐cc‐pVDZ‐F12 basis sets on the fluorine atoms and were performed on the corresponding minimum structures. This leads to negligible changes in vibrational frequencies for ${\mathrm{IF}}_{5}$ . For even larger molecules ($F_{2}\mathrm{CCO}$ , ${\mathrm{BMe}}_{3}$ , ${B(\mathrm{OH})}_{3}$ , ${\mathrm{AlMe}}_{3}$ , ${\mathrm{Al}(\mathrm{OH})}_{3}$ , ${\mathrm{CMe}}_{3}^{+}$ , ${\mathrm{SiMe}}_{3}^{+}$ ), structure optimization and harmonic vibrational frequency calculations were performed using the aug‐cc‐pVDZ‐F12 basis set on all atoms. Comparative calculations for the anchor system ${\mathrm{OCF}}_{2}$ suggest that the error introduced by this reduced basis is negligible (see Table S19 in Supporting Information). Thermal contributions to reaction enthalpies at 298.15 K were calculated using the standard rigid‐rotor‐harmonic‐oscillator approach. While the F12 basis sets for the coupled‐cluster calculations do already include scalar relativistic ECPs for the third‐row p‐block elements (Ga‐Br), the def2 basis sets used for the DFT computations (see below) include ECPs only starting with the fourth row, and computations for Ga‐Br therefore by default lack scalar relativistic effects. We therefore estimated the ECP effect for third‐row central atoms in the coupled‐cluster calculations from the difference between CCSD(T*)‐F12a calculations with aug‐cc‐pwCVTZ basis (without ECP) and aug‐cc‐pwCVTZ‐PP basis (with ECP) and back‐corrected the reference data for comparison with DFT methods. The original reference data including scalar relativistic effects are also provided in Supporting Information (Table S20).

Exploratory CCSD(T*)‐F12a calculations on the FIA of the larger Lewis acid ${B(C}_{6}F_{5}{)}_{3}$ (optimized at the BP86‐D4/def2‐TZVPD level) were performed using the PNO‐based implementation available in MOLPRO.^[38]^ To reduce computational cost, the aug‐cc‐pVTZ‐F12 basis set was only assigned to $F^{-}$ , B and the C atoms directly attached to the B atom (the respective DZ basis was employed for all other atoms). For ${\mathrm{BMe}}_{3}$ this approach results in very small deviations (1–2 kJ/mol, depending on PNO cutoffs) from the corresponding FIA71 reference value (see Table S14).

Additional higher‐order coupled‐cluster calculations were performed using the MRCC program, version 2018,[^39^, ^40^] the local natural orbital (LNO) ansatz^[41]^ for ${\mathrm{OCF}}_{2}$ and ${\mathrm{SiMe}}_{3}^{+}$ , and aug‐cc‐pVDZ basis sets.[^42^, ^43^]

DFT single‐point energy calculations were performed at the CCSD(T*)‐F12a/AVTZ‐F12 structures with a local version of Turbomole, based on release 7.8.[^44^, ^45^] For the evaluation of approximate exchange‐correlation (XC) functionals, extended def2‐QZVPD[^46^, ^47^] basis sets were used for all atoms, including the respective ECPs where appropriate. The calculations employed a fine integration grid (gridsize 5) and the multipole‐accelerated RI‐J approximation^[48]^ together with the corresponding auxiliary basis sets.^[49]^ The energy convergence criterion was set to $10^{-9}$ Hartree ($scfconv 9), the density convergence to $10^{-7}$ ($denconv 1.d‐7). In addition, a variety of smaller basis sets (def2‐SVP, def2‐SVPD, def2‐TZVP, def2‐TZVPP, def2‐TZVPD, def2‐QZVP and def2‐QZVPP)[^46^, ^47^] have been studied to understand basis‐set requirements. To evaluate the influence of the structure on the FIA, structure optimizations were also performed at various DFT levels with the same settings as for the energy calculations, using a small selection of XC‐functionals with convergence criteria of $10^{-7}$ a.u. for the energy and $10^{-4}$ for the Cartesian gradient norm. Harmonic vibrational frequencies were calculated to ensure that the structures converged into local minima and to obtain thermodynamic contributions that may be compared to the corresponding CCSD(T*)‐F12a/AVTZ‐F12 data.

A wide range of exchange‐correlation (XC) functionals from all five rungs of the usual ladder hierarchy^[50]^ was evaluated overall (52 functionals and four composite methods): LDA functional SVWN[^51^, ^52^] on rung 1, the rung 2 GGAs BP86,[^53^, ^54^, ^55^] B97‐D,^[56]^ PBE,[^57^, ^58^] and BLYP,[^53^, ^59^, ^60^] the meta‐GGA functionals TPSS,^[61]^ M06‐L,^[62]^ MN15‐L,^[63]^ and $r^{2}\mathrm{SCAN}$ ,[^64^, ^65^] the global hybrid functionals TPSSh,[^66^, ^67^] B3LYP,^[68]^ PBE0,^[69]^ M06,^[70]^ PW6B95,^[71]^ MN15,^[72]^ BHLYP,^[73]^ and M06‐2X,^[70]^ the range‐separated hybrids ω B97M‐V,^[74]^ ω B97X,^[75]^ ω B97X‐V,^[76]^ ω B97X‐D,^[77]^ and CAM‐B3LYP,^[78]^ the local hybrids LH07s‐SVWN,^[79]^ LH07t‐SVWN,^[80]^ LH12ct‐SsirPW92,^[81]^ LH12ct‐SsifPW92,^[81]^ LH14t‐calPBE,^[82]^ LH20t,^[83]^ LHJ14,^[84]^ LHJ‐HF,^[85]^ LHJ‐HFcal,^[85]^ mPSTS‐noa2,[^86^, ^87^] TMHF,^[85]^ TMHF‐3p,^[85]^ scLH22t,^[88]^ scLH22ta^[88]^ (the latter two contain strong‐correlation corrections), the range‐separated local hybrids ω LH22t^[89]^ and ω LH23tdE^[30]^ (the latter with strong‐correlation corrections), the double hybrid functionals B2PLYP,^[90]^ B2GP‐PLYP,^[91]^ B2K‐PLYP,^[92]^ B2T‐PLYP,^[92]^ PWPB95,^[93]^ PBE0‐DH,^[94]^ DSD‐BLYP,^[95]^ DSD‐PBEP86,^[96]^ DSD‐PBEB95,^[97]^ ${\mathrm{Pr}}^{2}\mathrm{SCAN}$ 50,^[98]^ ${\mathrm{Pr}}^{2}\mathrm{SCAN}$ 69,^[98]^ $\kappa {\mathrm{Pr}}^{2}\mathrm{SCAN}$ 50,^[98]^ and the range‐separated double hybrids $\omega {\mathrm{Pr}}^{2}\mathrm{SCAN}$ 50,^[98]^ and ω B97M(2).^[99]^ Grimme's “3c” composite methods PBEh‐3c,^[100]^ B97‐3c,^[101]^ $r^{2}\mathrm{SCAN}$ ‐3c,^[102]^ and ω B97X‐3c^[103]^ were also evaluated.

Computations with ω B97X‐3c and with the double hybrids were performed with the ORCA program,[^104^, ^105^] release 6.0.0, with the convergence criterion VeryTightSCF, the large grid DEFGRID3 and the RIJCOSX^[106]^ approximation. The RI approximation was also applied to the MP2 part of the double hybrids using the appropriate auxiliary basis sets.[^49^, ^107^, ^108^] Where indicated, D3 dispersion corrections with Becke and Johnson damping,[^109^, ^110^] D3(BJ), or D4 dispersion corrections^[111]^ were included.

For the anchor‐point computations, we used the present CCSD(T*)‐F12a FIA values of 211.8 kJ/mol and 953.8 kJ/mol for the ${\mathrm{OCF}}_{2}$ and ${\mathrm{SiMe}}_{3}^{+}$ system, respectively (with electronic contributions of 211.7 kJ/mol and 960.2 kJ/mol; see Table S1). The ${\mathrm{OCF}}_{2}$ value is expected to be more accurate than the previously used experimental FIAs of 208.8 kJ/mol^[6]^ but is clearly within its error margin. No experimental value is available for ${\mathrm{SiMe}}_{3}^{+}$ . Our value for that system is close to the computed CCSD(T)/CBS(T,Q) value of 952.5 kJ/mol from Ref. [12].

## Conflict of Interests

The authors declare no conflict of interest.

1

## Supporting information

As a service to our authors and readers, this journal provides supporting information supplied by the authors. Such materials are peer reviewed and may be re‐organized for online delivery, but are not copy‐edited or typeset. Technical support issues arising from supporting information (other than missing files) should be addressed to the authors.

Supporting Information

## Data Availability

The data that support the findings of this study are available in the supplementary material of this article.
