# Supplementary material for: The Fluoride Ion Affinity Revisited: Do We Need the Anchor‐Point Approach?
Source: Chemistry. 2025 Apr 22;31(26):e202404662. doi: 10.1002/chem.202404662 (PMC12063057; doi:10.1002/chem.202404662)
Supplement: Supplementary file 1 — Supporting Information [file CHEM-31-e202404662-s001.pdf]

Chemistry–A European Journal

Supporting Information

The Fluoride Ion Affinity Revisited: Do We Need the Anchor-Point Approach?

Morten Lehmann, Salimot Natalie Balogun, Marc Reimann,* and Martin Kaupp*

Supporting Information:

The Fluoride Ion Affinity Revisited: Do We Need the Anchor-Point Approach?

Morten Lehmann,[a] Salimot N. Balogun, [a] Marc Reimann,*[a,b] and Martin Kaupp*[a]

[a] *Technische Universität Berlin, Institut für Chemie, D-10623 Berlin, Germany*

[b] *Universität Innsbruck, Institut für Ionenphysik und Angewandte Physik, A-6020 Innsbruck, Austria*

E-Mail: marc.reimann@uibk.ac.at; martin.kaupp@tu-berlin.de

Table S1: Full benchmark reaction energies and resulting FIAs in kJ/mol of FIA71 at CCSD(T*)-F12a/AVTZ-F12//CCSD(T*)-F12a/AVTZ-F12 level.

LA	ΔE^{FIA}	FIA	LA	ΔE^{FIA}	FIA
F_2	101.4	100.6	SO_2	225.5	222.7
HF	185.0	189.9	SO_3	377.4	373.2
H_2O	114.7	116.3	SeO_2[a]	270.4	267.8
H_2CCO	163.7	159.6	TeO_2	337.4	334.7
H_2CO	97.4	94.2	OCF_2	211.7	211.8
HCO_2H	89.3	89.1	NOF	91.6	90.3
HCN	195.3	195.1	NO_2F	86.3	86.6
BH_3	280.9	277.7	OPF_3	243.1	242.1
BF_3	345.0	344.2	SOF_2	180.1	179.7
BF_2Cl	373.9	372.2	SOF_4	262.4	261.3
$BFCl_2$	391.7	389.4	SO_2F_2	168.3	167.9
BCl_3	401.7	398.9	BeF_2	372.8	371.2
BBr_3[a]	428.7	425.1	PF_3	199.8	199.1
AlH_3	386.3	384.5	PF_5	379.6	378.3
AlF_3	483.5	481.3	AsF_3[a]	243.9	243.0
AlF_2Cl	494.2	491.7	AsF_5[a]	436.7	434.7
$AlFCl_2$	501.5	498.8	SbF_3	292.3	291.0
$AlCl_3$	506.2	503.4	SbF_5	494.8	492.8
GaF_3[a]	452.2	450.4	SF_2	197.0	196.2
$GaCl_3$[a]	438.1	435.7	SF_4	220.7	221.1
InF_3	437.1	435.4	SeF_4[a]	293.1	292.6
$InCl_3$	420.5	418.3	TeF_4	350.9	349.9
H_3C^+	1093.9	1079.0	ClF_3	244.2	243.9
F_3C^+	1094.1	1091.7	ClF_5	198.6	202.2
H_3Si^+	1096.4	1088.0	BrF_3[a]	286.9	286.2
SiH_4	171.0	171.8	BrF_5[a]	245.8	247.9
SiF_4	305.4	304.4	IF_3	318.4	317.9
$SiCl_4$	328.1	325.6	IF_5	272.9	273.6
F_3Si^+	1247.1	1243.2	F_2CCO[b]	262.4	260.6
GeF_4[a]	353.6	352.1	BMe_3[b]	247.4	248.2
$GeCl_4$[a]	305.2	302.9	BOH_3[b]	185.2	187.6
SnF_4	411.7	410.0	$AlMe_3$[b]	367.3	368.6
$SnCl_4$	359.1	356.9	$AlOH_3$[b]	376.8	375.8
BeO	578.3	573.4	Me_3C^+[b]	829.9	818.0
CO	44.8	45.1	Me_3Si^+[b]	960.2	953.8
CO_2	136.6	136.2			

[a]Back-corrected by subtracting the effect of scalar relativistic ECPs in the benchmark data for comparison with non-relativistic DFT computations, see text. [b]CCSD(T*)-F12a/AVTZ-F12//CCSD(T*)-F12a/AVDZ-F12.

Table S2: Estimate of remaining basis-set and correlation errors in kJ/mol in the benchmark ΔE^{FIA} values for selected systems (see discussion in main text).

	ΔE^{FIA} benchmark value[a]	$\delta^{QZ\text{-}TZ}$ [b]	$\delta^{T\text{-}(T)}$ [c]	$\delta^{Q\text{-}(T)}$ [d]
OCF_2	211.7	+0.1	−0.4	–
$SiMe_3^+$	960.2	+0.2	−0.5	–
BeO	578.3	+0.7	−1.8	−2.4
F_2	101.4	−2.9	−2.7	+4.2

[a]CCSD(T*)-F12/aug-cc-pVTZ-F12 result. [b]Difference from CCSD(T*)-F12/aug-cc-pVQZ-F12 result. [c]Difference between CCSDT/aug-cc-pVDZ and CCSD(T)/aug-cc-pVDZ results. The LNO approach was employed for OCF_2 and $SiMe_3^+$ due to their size. [d]Difference between CCSDTQ/aug-cc-pVDZ and CCSD(T)/aug-cc-pVDZ results.

Table S3: ΔE^{FIA} MAE in kJ/mol obtained with the CCSD(T) reference structures compared to structures reoptimized with the corresponding functional used in the energy calculations.

structure	BP86-D4	TPSS-D4	r²SCAN-D4	TPSSh-D4	PW6B95-D4
CCSD(T)	25.4	17.9	11.9	11.9	6.7
DFT-optimized	24.7	17.7	12.2	12.0	6.6
	LH20t-D4	PBEh-3c	B97-3c	r²SCAN-3c	
CCSD(T)	5.8	142.0	34.9	44.3	
DFT-optimized	5.7	143.0	37.7	46.8	

Table S4: ΔE^{FIA} MAE in kJ/mol for LH20t-D4 comparing structures optimized at different levels.

structure	CCSD(T)	BP86-D4/def2-QZVPD	BP86-D4/def2-TZVPD	BP86-D4/def2-TZVP
	5.8	5.9	5.9	5.9
structure	BP86-D4/def2-SVPD	BP86-D4/def2-SVP	TPSS-D4	r²SCAN-D4
	7.0	7.2	5.6	5.7
structure	TPSSh-D4	PW6B95-D4	LH20t-D4	
	5.7	5.7	5.7	
structure	PBEh-3c	B97-3c	r²SCAN-3c	
	5.9	5.9	5.7	

Table S5: MAE of full FIAs in kJ/mol for LH20t-D4 comparing structures optimized at different levels.

structure		BP86-D4/def2-QZVPD	BP86-D4/def2-TZVPD	BP86-D4/def2-TZVP
		5.6	5.6	5.6
structure	BP86-D4/def2-SVPD	BP86-D4/def2-SVP	TPSS-D4	r²SCAN-D4
	6.7	7.0	5.4	5.5
structure	TPSSh-D4	PW6B95-D4	LH20t-D4	
	5.5	5.7	5.8	
structure	PBEh-3c	B97-3c	r²SCAN-3c	
	6.0	5.7	5.6	

Table S6: Directly calculated reaction energies ΔE^{FIA} in kJ/mol obtained with different XC functionals using structures optimized with the corresponding functional. Except for the composite methods, def2-QZVPD basis sets have been used.

LA	BP86-D4	TPSS-D4	R^2SCAN-D4	TPSSh-D4	PW6B95-D4
F_2	176.4	175.1	166.7	149.1	110.5
HF	187.8	188.3	194.6	189.0	185.4
H_2O	116.9	116.6	121.2	116.4	112.6
H_2CCO	158.0	161.7	174.9	166.4	162.8
H_2CO	113.5	115.7	123.9	114.7	106.1
HCO_2H	89.2	93.0	102.5	94.1	88.8
HCN	192.2	187.0	202.0	191.7	193.4
BH_3	280.4	282.6	295.7	286.9	291.7
BF_3	314.2	327.2	342.3	334.8	340.9
BF_2Cl	344.1	357.0	373.1	365.3	369.5
$BFCl_2$	362.5	375.4	391.3	384.0	386.6
BCl_3	373.0	385.9	400.9	394.7	395.8
BBr_3	400.7	411.4	429.8	420.9	422.2
AlH_3	364.2	374.0	393.0	380.6	382.9
AlF_3	450.6	459.5	483.7	468.0	478.5
AlF_2Cl	459.8	470.5	493.2	479.2	486.8
$AlFCl_2$	466.5	478.6	499.7	487.3	492.4
$AlCl_3$	471.1	484.4	503.8	493.1	496.2
GaF_3	415.2	422.6	448.4	431.6	440.9
$GaCl_3$	404.3	416.7	437.0	424.9	424.2
InF_3	398.6	404.6	428.5	413.1	422.1
$InCl_3$	388.3	399.4	419.4	406.7	405.5
H_3C^+	1103.6	1101.7	1119.0	1105.8	1109.3
F_3C^+	1050.4	1061.7	1086.7	1075.5	1085.9
H_3Si^+	1070.9	1086.9	1107.2	1095.0	1094.4
SiH_4	162.0	169.9	178.6	174.0	176.0
SiF_4	281.6	295.4	307.6	301.0	308.2
$SiCl_4$	304.6	319.3	328.9	324.4	324.6
F_3Si^+	1200.3	1215.9	1244.5	1230.1	1242.9
GeF_4	328.6	339.8	358.5	345.6	351.4
$GeCl_4$	290.2	304.2	313.5	307.5	303.6
SnF_4	382.3	391.2	415.4	398.2	405.0
$SnCl_4$	335.1	349.4	362.6	353.2	348.8
BeO	553.3	561.5	581.2	571.4	579.5
CO	70.6	71.9	73.3	67.8	55.7
CO_2	132.6	135.9	148.8	140.8	139.9
SO_2	223.3	229.4	242.6	235.1	233.0
SO_3	340.6	348.8	374.0	363.6	374.6
SeO_2	267.2	275.0	287.9	283.4	285.0
TeO_2	320.3	331.5	346.5	342.8	346.7
OCF_2	200.5	206.7	222.6	211.0	209.8
NOF	128.5	131.9	133.2	122.4	104.8
NO_2F	117.5	120.1	125.9	111.6	95.8
OPF_3	227.4	239.9	248.1	244.2	247.3
SOF_2	193.2	196.2	200.8	194.0	187.6
SOF_4	250.0	260.3	273.2	263.6	264.1
SO_2F_2	169.0	176.9	183.0	177.3	173.5
BeF_2	346.9	355.3	373.5	362.1	369.5
PF_3	197.2	205.1	210.6	206.2	205.2
PF_5	347.0	364.1	378.8	372.0	380.7
AsF_3	238.6	245.3	252.4	247.0	246.3
AsF_5	401.7	415.8	437.7	424.8	433.2
SbF_3	276.5	284.3	295.2	287.8	288.6
SbF_5	452.6	465.0	491.5	475.3	484.8
SF_2	211.7	215.8	219.4	212.8	204.2
SF_4	238.2	241.1	246.9	237.5	229.2
SeF_4	298.5	302.6	309.6	302.4	299.3
TeF_4	340.1	346.8	356.9	350.0	350.4
ClF_3	280.4	281.2	281.4	269.2	248.4
ClF_5	280.7	272.1	260.9	242.3	203.3
BrF_3	311.7	311.9	313.4	305.1	292.8
BrF_5	318.5	307.3	296.4	285.5	260.0
IF_3	331.8	332.5	334.3	329.4	322.9
IF_5	323.3	309.1	295.6	292.4	284.3
F_2CCO	255.4	262.8	279.7	267.3	263.7
BMe_3	228.3	241.8	244.6	245.3	244.2
BOH_3	160.8	171.0	180.3	175.5	176.4
$AlMe_3$	337.7	353.0	364.7	358.7	357.8
$AlOH_3$	348.0	357.3	374.7	363.5	368.3
Me_3C^+	784.9	799.2	810.7	809.0	810.0
Me_3Si^+	914.6	935.3	951.8	945.4	945.4

Table S6 continued.

LA	LH20t-D4	PBEh-3c	B97-3c	r^2SCAN-3c
F_2	95.2	184.9	208.1	198.4
HF	186.3	279.0	215.7	217.0
H_2O	112.7	187.7	139.1	139.9
H_2CCO	167.5	323.4	199.3	212.1
H_2CO	105.0	217.3	147.5	150.8
HCO_2H	88.8	202.8	124.0	131.9
HCN	192.8	320.8	228.5	242.2
BH_3	276.5	427.8	322.3	325.1
BF_3	341.2	494.9	365.1	377.6
BF_2Cl	372.5	532.7	399.6	412.9
$BFCl_2$	391.4	558.7	420.5	434.0
BCl_3	402.0	575.3	432.3	445.8
BBr_3	427.4	604.1	455.8	479.1
AlH_3	382.1	546.4	407.0	411.7
AlF_3	479.4	623.5	505.1	517.2
AlF_2Cl	489.4	646.5	519.2	529.6
$AlFCl_2$	496.0	666.3	529.5	538.0
$AlCl_3$	501.0	683.2	536.6	543.6
GaF_3	445.2	592.8	464.3	484.8
$GaCl_3$	429.8	613.4	461.8	479.8
InF_3	428.7	568.7	450.5	468.5
$InCl_3$	411.9	589.4	445.2	465.3
H_3C^+	1088.8	1275.9	1153.8	1156.5
F_3C^+	1092.2	1283.3	1110.2	1129.4
H_3Si^+	1091.2	1274.2	1117.7	1141.2
SiH_4	165.4	290.6	187.1	192.3
SiF_4	301.0	457.6	330.4	344.7
$SiCl_4$	326.0	496.1	358.9	370.2
F_3Si^+	1241.5	1411.9	1257.0	1281.7
GeF_4	347.5	485.5	374.2	391.6
$GeCl_4$	302.3	479.6	341.6	354.7
SnF_4	405.7	538.4	435.6	452.8
$SnCl_4$	347.9	525.2	390.2	407.4
BeO	584.3	747.8	596.7	621.2
CO	54.6	167.5	103.5	106.4
CO_2	145.4	291.7	173.4	184.1
SO_2	240.0	366.6	255.0	263.9
SO_3	382.1	520.5	367.0	383.3
SeO_2	294.3	416.7	294.4	306.5
TeO_2	358.2	471.5	343.1	362.0
OCF_2	214.6	352.3	242.6	257.9
NOF	97.6	186.1	163.0	162.6
NO_2F	93.8	196.6	155.7	156.1
OPF_3	242.3	399.9	273.3	281.4
SOF_2	185.3	316.1	239.1	240.9
SOF_4	263.0	419.4	308.4	311.5
SO_2F_2	170.6	331.2	224.1	228.1
BeF_2	368.3	522.6	383.9	404.3
PF_3	201.0	332.5	235.6	240.5
PF_5	379.6	532.3	404.9	411.9
AsF_3	244.2	364.9	276.1	283.7
AsF_5	433.7	567.4	450.1	466.6
SbF_3	289.4	413.1	325.7	336.1
SbF_5	488.9	623.6	506.7	529.6
SF_2	202.5	320.3	248.5	250.9
SF_4	227.7	362.5	292.4	290.5
SeF_4	298.7	421.4	342.6	347.2
TeF_4	352.2	480.5	392.2	401.8
ClF_3	247.6	357.7	336.4	325.8
ClF_5	201.2	318.1	364.2	336.4
BrF_3	294.1	401.0	359.6	357.5
BrF_5	259.9	365.6	377.6	359.8
IF_3	326.7	442.6	385.6	385.9
IF_5	282.2	409.4	395.3	374.9
F_2CCO	266.7	420.7	298.8	318.0
BMe_3	245.3	409.0	271.8	285.6
BOH_3	172.2	305.3	205.6	213.6
$AlMe_3$	359.6	531.6	382.8	396.5
$AlOH_3$	364.0	514.8	393.7	408.7
Me_3C^+	820.4	1004.0	836.7	857.5
Me_3Si^+	948.1	1139.0	963.7	991.5

Table S7: Directly calculated reaction energies ΔE^{FIA} in kJ/mol at LH20t-D4 level using structures optimized at different levels. Where def2-QZVPD basis sets have been used, the basis-set label is omitted.

LA	BP86-D4/def2-QZVPD	BP86-D4/def2-TZVPD	BP86-D4/def2-TZVP	BP86-D4/def2-SVPD	BP86-D4/def2-SVP
F_2	93.9	93.8	93.6	91.4	92.3
HF	186.3	186.5	186.4	186.4	186.2
H_2O	111.8	111.6	110.1	111.9	104.8
H_2CCO	165.8	165.8	166.2	166.6	168.0
H_2CO	103.5	103.5	103.8	103.9	99.7
HCO_2H	86.4	86.5	87.0	86.8	86.9
HCN	191.1	190.4	191.9	189.6	194.4
BH_3	276.2	276.3	276.1	276.7	274.7
BF_3	340.7	340.8	341.0	340.6	341.4
BF_2Cl	372.0	372.0	372.1	372.1	372.6
$BFCl_2$	390.9	390.8	390.9	390.9	391.4
BCl_3	401.5	401.4	401.4	401.5	402.0
BBr_3	426.7	426.6	426.6	426.6	427.0
AlH_3	382.0	382.0	381.9	380.2	381.5
AlF_3	479.5	479.4	479.4	479.4	481.0
AlF_2Cl	489.3	489.3	489.3	488.9	490.3
$AlFCl_2$	495.9	495.8	495.8	495.1	496.4
$AlCl_3$	500.7	500.7	500.6	499.5	500.8
GaF_3	444.6	444.5	444.5	444.9	446.5
$GaCl_3$	429.3	429.2	429.1	429.1	430.3
InF_3	428.3	428.2	428.3	428.6	431.3
$InCl_3$	411.4	411.3	411.3	411.6	413.2
H_3C^+	1088.3	1088.2	1088.2	1088.6	1087.6
F_3C^+	1091.6	1091.8	1091.8	1091.7	1092.7
H_3Si^+	1090.9	1090.8	1090.8	1086.4	1088.6
SiH_4	164.8	164.7	164.7	161.8	161.6
SiF_4	300.5	300.6	300.7	304.8	305.4
$SiCl_4$	325.0	325.0	324.9	322.4	323.3
F_3Si^+	1241.6	1241.5	1241.5	1240.5	1242.3
GeF_4	346.5	346.6	346.5	346.9	349.2
$GeCl_4$	300.7	300.6	300.5	300.0	301.5
SnF_4	404.8	404.6	404.6	405.7	409.3
$SnCl_4$	346.6	346.3	346.5	346.3	348.6
BeO	584.9	585.0	585.5	587.6	588.3
CO	54.3	54.3	53.9	54.6	51.8
CO_2	143.3	143.4	143.9	144.4	146.0
SO_2	237.5	237.3	236.7	235.5	240.1
SO_3	381.5	381.4	381.8	376.6	381.8
SeO_2	292.3	292.0	291.5	288.8	293.6
TeO_2	357.2	356.5	356.4	351.8	358.4
OCF_2	212.5	212.7	212.9	212.6	214.8
NOF	98.3	98.3	97.6	97.0	94.5
NO_2F	92.0	91.9	93.0	91.6	93.8
OPF_3	241.0	241.3	241.4	245.3	246.4
SOF_2	182.9	182.8	182.5	186.1	188.2
SOF_4	259.3	259.4	259.5	264.3	271.3
SO_2F_2	167.6	167.8	168.1	173.1	174.9
BeF_2	368.2	368.3	368.3	368.3	368.5
PF_3	199.0	199.1	198.6	201.3	203.2
PF_5	378.1	378.3	378.5	382.0	387.5
AsF_3	242.5	242.4	242.1	242.4	245.3
AsF_5	432.2	432.3	432.5	431.6	436.4
SbF_3	287.8	287.6	287.6	288.3	292.9
SbF_5	488.0	488.0	488.1	489.4	494.8
SF_2	200.5	200.4	199.7	201.2	202.7
SF_4	224.6	224.6	224.1	228.5	231.8
SeF_4	296.9	296.9	296.6	298.5	302.7
TeF_4	350.7	350.7	350.7	353.3	359.9
ClF_3	247.8	247.8	247.1	248.7	249.4
ClF_5	206.6	207.0	206.7	218.3	215.1
BrF_3	294.4	294.5	294.1	298.1	298.9
BrF_5	265.2	265.8	265.4	275.5	272.8
IF_3	326.7	327.1	326.8	333.7	336.4
IF_5	279.3	281.0	280.2	300.9	301.7
F_2CCO	264.7	264.7	264.5	266.1	268.0
BMe_3	245.0	245.0	245.0	244.8	244.5
BOH_3	171.4	171.3	171.2	171.1	168.9
$AlMe_3$	359.2	359.1	359.2	357.6	358.9
$AlOH_3$	363.9	363.7	363.2	362.7	360.2
Me_3C^+	820.6	820.6	820.7	821.0	820.9
Me_3Si^+	947.6	947.5	947.5	944.1	945.6

Table S7 continued.

LA	TPSS-D4	r^2SCAN-D4	TPSSh-D4	PW6B95-D4	LH20t-D4	PBEh-3c	B97-3c	r^2SCAN-3c
F_2	95.1	94.6	95.3	95.2	95.2	94.0	92.6	94.0
HF	186.2	186.4	186.3	186.3	186.3	186.6	186.4	186.4
H_2O	112.3	112.1	112.5	112.7	112.7	108.9	112.1	111.2
H_2CCO	166.0	167.1	167.0	167.5	167.5	167.0	166.4	166.7
H_2CO	103.7	104.6	104.6	104.9	105.0	101.5	104.0	103.9
HCO_2H	86.9	88.3	88.3	88.8	88.8	87.7	87.2	87.0
HCN	191.6	192.0	192.3	192.8	192.8	192.5	192.9	192.6
BH_3	276.4	276.3	276.5	276.4	276.5	275.1	276.0	276.3
BF_3	341.0	341.2	341.1	341.2	341.2	341.1	340.8	341.1
BF_2Cl	372.3	372.5	372.5	372.5	372.5	372.1	372.0	372.0
$BFCl_2$	391.1	391.3	391.3	391.4	391.4	390.9	390.8	390.8
BCl_3	401.6	401.9	401.9	402.0	402.0	401.5	401.3	401.3
BBr_3	426.9	427.1	427.2	427.3	427.4	427.3	426.6	426.6
AlH_3	382.1	382.2	382.2	382.2	382.1	381.6	381.7	381.9
AlF_3	479.6	479.5	479.5	479.4	479.4	479.4	479.2	479.6
AlF_2Cl	489.4	489.4	489.4	489.3	489.4	489.4	489.1	489.4
$AlFCl_2$	496.0	496.0	496.0	496.0	496.0	496.0	495.8	495.9
$AlCl_3$	500.8	501.0	501.0	500.9	501.0	500.9	500.7	500.6
GaF_3	445.0	445.3	445.2	445.2	445.2	444.7	444.9	445.3
$GaCl_3$	429.5	429.8	429.7	429.8	429.8	429.2	429.4	429.8
InF_3	428.6	428.8	428.7	428.7	428.7	428.2	428.3	428.8
$InCl_3$	411.6	411.9	411.8	411.9	411.9	412.0	411.1	411.7
H_3C^+	1088.3	1088.7	1088.7	1088.7	1088.8	1088.1	1088.5	1088.3
F_3C^+	1091.8	1092.2	1092.2	1092.1	1092.2	1092.3	1092.1	1091.9
H_3Si^+	1091.0	1091.3	1091.2	1091.3	1091.2	1089.9	1090.7	1090.9
SiH_4	165.2	165.2	165.4	165.4	165.4	163.6	164.1	165.0
SiF_4	300.9	301.0	301.1	301.0	301.0	303.9	300.3	300.9
$SiCl_4$	325.5	325.8	325.8	326.0	326.0	325.2	323.9	324.4
F_3Si^+	1241.7	1241.6	1241.7	1241.5	1241.5	1241.3	1241.1	1241.6
GeF_4	347.2	347.5	347.5	347.5	347.5	347.3	346.2	347.1
$GeCl_4$	301.5	302.1	302.1	302.3	302.3	301.8	300.1	301.1
SnF_4	405.4	405.8	405.7	405.7	405.7	405.7	403.8	405.4
$SnCl_4$	347.3	347.7	347.7	347.9	347.9	348.4	345.2	346.6
BeO	584.8	584.7	584.5	584.3	584.3	584.6	586.1	585.8
CO	54.3	54.5	54.5	54.5	54.6	51.1	54.1	54.2
CO_2	143.6	144.9	144.9	145.4	145.4	145.1	143.8	144.2
SO_2	238.8	239.7	239.8	239.9	240.0	240.2	235.7	237.2
SO_3	382.2	382.5	382.4	382.1	382.1	381.3	377.9	380.1
SeO_2	293.7	294.5	294.4	294.4	294.3	294.2	290.2	292.2
TeO_2	358.3	358.8	358.5	358.2	358.2	358.7	354.0	356.3
OCF_2	213.1	214.3	214.2	214.6	214.6	214.3	212.9	213.5
NOF	98.7	98.6	98.2	97.5	97.6	92.3	96.9	97.8
NO_2F	91.9	93.4	92.7	93.3	93.8	90.7	91.4	91.6
OPF_3	241.8	242.2	242.2	242.3	242.3	246.1	240.9	241.6
SOF_2	184.5	184.9	185.2	185.3	185.3	185.5	181.7	183.6
SOF_4	261.4	262.3	262.6	262.9	263.0	265.6	259.0	259.4
SO_2F_2	169.2	169.9	170.3	170.6	170.6	173.3	169.2	169.1
BeF_2	368.3	368.2	368.3	368.2	368.3	368.7	367.8	368.5
PF_3	200.4	200.9	200.9	201.0	201.0	202.1	197.1	198.7
PF_5	379.1	379.5	379.5	379.6	379.6	381.6	378.8	378.8
AsF_3	243.9	244.2	244.2	244.2	244.2	243.1	240.9	243.3
AsF_5	433.3	433.7	433.6	433.7	433.7	433.8	431.2	432.6
SbF_3	289.2	289.4	289.5	289.4	289.4	289.2	287.2	289.3
SbF_5	488.8	489.1	489.0	489.0	488.9	489.2	487.9	488.8
SF_2	201.6	202.1	202.3	202.5	202.5	203.0	198.0	199.7
SF_4	226.6	227.1	227.5	227.7	227.7	229.6	222.6	224.7
SeF_4	298.3	298.6	298.7	298.7	298.7	298.7	294.3	297.3
TeF_4	352.0	352.3	352.3	352.3	352.2	353.1	349.7	352.6
ClF_3	247.0	246.9	247.3	247.6	247.6	248.3	248.7	245.7
ClF_5	201.4	201.0	200.4	201.2	201.2	203.0	218.7	206.4
BrF_3	293.9	293.6	294.0	294.1	294.1	293.7	292.9	293.2
BrF_5	261.3	259.5	259.9	259.9	259.9	260.0	264.5	262.2
IF_3	326.6	326.5	326.7	326.7	326.7	327.7	326.7	327.9
IF_5	276.6	281.7	281.7	281.7	282.2	278.8	282.5	281.5
F_2CCO	264.1	265.6	265.6	266.5	266.7	266.6	264.9	264.7
BMe_3	244.9	245.4	245.2	245.4	245.3	244.5	245.3	245.6
BOH_3	171.6	172.2	172.0	172.2	172.2	171.7	171.4	171.8
$AlMe_3$	359.3	359.5	359.4	359.5	359.6	359.4	359.4	359.4
$AlOH_3$	363.8	364.0	364.0	364.0	364.0	363.1	364.1	364.2
Me_3C^+	819.9	821.2	820.4	820.8	820.4	820.1	821.4	821.0
Me_3Si^+	947.7	948.2	947.9	948.1	948.1	947.9	947.9	948.1

Table S8: Directly calculated full FIA in kJ/mol at LH20t-D4 level using structures optimized at different levels. Where def2-QZVPD basis sets have been used, the basis-set label is omitted.

LA	BP86-D4/def2-QZVPD	BP86-D4/def2-TZVPD	BP86-D4/def2-TZVP	BP86-D4/def2-SVPD	BP86-D4/def2-SVP
F_2	93.8	93.8	93.4	91.3	91.9
HF	188.9	188.6	187.7	188.7	186.9
H_2O	115.2	115.0	114.0	115.0	110.0
H_2CCO	162.8	162.7	162.8	163.3	163.8
H_2CO	101.4	101.3	101.7	101.5	98.7
HCO_2H	87.3	87.3	87.7	87.4	87.6
HCN	194.0	193.7	194.0	192.9	195.6
BH_3	274.0	273.9	273.4	273.8	272.2
BF_3	340.5	340.6	340.5	340.3	340.2
BF_2Cl	370.7	370.6	370.5	370.6	370.4
$BFCl_2$	388.9	388.8	388.7	388.9	388.7
BCl_3	399.1	399.0	399.0	399.1	399.1
BBr_3	423.4	423.3	423.3	423.4	423.4
AlH_3	380.7	380.5	380.4	378.4	379.4
AlF_3	477.5	477.5	477.4	477.5	478.4
AlF_2Cl	487.0	487.0	486.9	486.6	487.5
$AlFCl_2$	493.4	493.3	493.3	492.6	493.4
$AlCl_3$	498.0	498.0	497.9	496.8	497.8
GaF_3	443.0	442.8	442.8	443.2	444.4
$GaCl_3$	427.0	426.9	426.9	426.8	427.7
InF_3	426.7	426.6	426.6	426.9	429.3
$InCl_3$	409.3	409.2	409.2	409.5	410.9
H_3C^+	1074.5	1074.4	1074.4	1074.8	1073.6
F_3C^+	1090.5	1090.6	1090.6	1090.7	1090.7
H_3Si^+	1083.2	1083.1	1083.1	1078.9	1080.6
SiH_4	166.3	166.0	165.9	162.8	163.8
SiF_4	299.8	299.8	299.8	303.4	303.6
$SiCl_4$	322.8	322.7	322.6	320.1	320.8
F_3Si^+	1237.9	1237.9	1237.8	1237.0	1238.3
GeF_4	345.2	345.4	345.2	345.5	347.4
$GeCl_4$	298.6	298.5	298.4	297.9	299.2
SnF_4	403.3	403.1	403.1	404.2	407.4
$SnCl_4$	344.5	344.3	344.4	344.2	346.3
BeO	580.4	580.5	580.9	582.5	583.0
CO	54.7	54.7	54.1	54.9	51.7
CO_2	143.8	144.0	144.1	144.9	145.2
SO_2	235.4	235.2	234.4	233.4	237.5
SO_3	378.1	378.1	378.2	373.5	377.8
SeO_2	290.4	290.1	289.5	287.0	291.3
TeO_2	355.1	354.5	354.3	349.8	356.0
OCF_2	213.4	213.6	213.4	213.5	214.2
NOF	97.3	97.2	96.5	96.0	93.2
NO_2F	92.1	92.0	92.8	91.7	93.3
OPF_3	240.4	240.8	240.7	244.2	244.7
SOF_2	182.6	182.4	182.0	185.7	187.3
SOF_4	259.0	259.0	258.9	263.3	269.4
SO_2F_2	167.5	167.8	167.9	172.7	173.8
BeF_2	367.0	366.9	366.9	367.1	366.5
PF_3	198.7	198.8	198.2	200.8	202.1
PF_5	377.2	377.4	377.4	380.4	384.6
AsF_3	241.7	241.7	241.3	241.5	244.1
AsF_5	430.6	430.7	430.7	429.9	434.0
SbF_3	286.8	286.6	286.5	287.2	291.4
SbF_5	486.3	486.2	486.2	487.5	492.4
SF_2	199.8	199.6	199.0	200.5	201.7
SF_4	224.9	224.9	224.4	228.7	231.5
SeF_4	296.4	296.4	296.1	297.8	301.6
TeF_4	349.9	349.8	349.8	352.2	358.5
ClF_3	247.2	247.2	246.4	248.1	248.4
ClF_5	208.2	208.6	208.1	219.3	216.0
BrF_3	293.6	293.8	293.3	297.2	297.9
BrF_5	266.3	266.9	266.4	276.1	273.5
IF_3	325.9	326.3	326.1	332.8	335.2
IF_5	280.7	282.3	281.5	301.4	302.1
F_2CCO	263.8	263.9	263.5	265.0	265.9
BMe_3	246.3	245.7	245.4	245.6	244.7
BOH_3	171.1	170.9	170.5	170.8	167.8
$AlMe_3$	360.8	360.3	360.1	358.7	359.4
$AlOH_3$	370.3	370.2	370.1	368.3	366.4
Me_3C^+	810.0	809.3	809.2	809.9	808.9
Me_3Si^+	941.5	941.0	940.9	937.7	938.6

Table S8 continued.

LA	TPSS-D4	r^2SCAN-D4	TPSSh-D4	PW6B95-D4	LH20t-D4	PBEh-3c	B97-3c	r^2SCAN-3c
F_2	95.0	94.7	95.3	95.5	95.7	93.8	92.7	94.1
HF	189.3	189.1	189.6	190.2	190.4	184.5	189.2	188.0
H_2O	115.0	115.3	114.9	114.6	114.1	111.4	114.8	114.7
H_2CCO	163.0	163.6	163.8	163.8	163.7	161.8	163.2	162.9
H_2CO	101.4	102.2	102.1	102.2	101.9	98.4	101.5	100.9
HCO_2H	87.7	88.9	88.9	89.1	88.8	87.3	87.7	87.3
HCN	194.5	194.3	194.5	194.1	194.0	192.3	194.2	193.8
BH_3	273.9	273.7	273.8	273.4	273.4	271.2	273.7	273.0
BF_3	340.6	340.8	340.6	340.5	340.3	339.6	340.4	340.8
BF_2Cl	370.8	370.9	370.9	370.9	370.7	369.8	370.4	370.4
$BFCl_2$	389.0	389.2	389.2	389.1	389.0	388.0	388.5	388.5
BCl_3	399.3	399.4	399.4	399.4	399.4	398.3	398.8	398.7
BBr_3	423.6	423.7	423.8	423.8	423.8	423.5	423.1	423.0
AlH_3	380.6	380.6	380.7	380.7	380.5	378.9	380.2	379.4
AlF_3	477.5	477.2	477.5	477.3	477.3	477.1	477.1	477.5
AlF_2Cl	487.1	486.9	487.1	487.0	487.0	486.7	486.8	487.0
$AlFCl_2$	493.4	493.3	493.4	493.4	493.4	493.0	493.2	493.2
$AlCl_3$	498.1	498.1	498.2	498.2	498.2	497.7	497.9	497.8
GaF_3	443.3	443.4	443.4	443.5	443.3	442.7	443.2	443.5
$GaCl_3$	427.2	427.4	427.4	427.4	427.4	426.4	427.1	427.4
InF_3	427.0	427.1	427.1	427.1	427.1	426.3	426.5	427.1
$InCl_3$	409.5	409.8	409.7	409.8	409.8	409.5	409.0	409.5
H_3C^+	1074.3	1074.2	1074.2	1073.5	1073.4	1073.0	1074.1	1074.2
F_3C^+	1090.6	1090.7	1090.7	1090.4	1090.2	1089.6	1090.8	1090.6
H_3Si^+	1083.1	1083.1	1083.2	1083.0	1082.9	1081.4	1082.7	1082.7
SiH_4	166.5	166.5	166.5	166.2	166.4	163.9	166.1	165.3
SiF_4	300.1	300.1	300.2	300.0	299.8	302.1	299.6	299.9
$SiCl_4$	323.1	323.4	323.4	323.5	323.5	322.4	321.7	322.0
F_3Si^+	1238.0	1237.8	1237.9	1237.7	1237.8	1237.5	1237.4	1237.8
GeF_4	345.9	346.1	346.2	346.1	345.9	345.5	344.9	345.6
$GeCl_4$	299.3	299.8	299.8	299.9	299.9	299.2	298.0	298.9
SnF_4	403.8	404.1	404.1	404.1	404.0	403.9	402.3	403.7
$SnCl_4$	345.2	345.5	345.6	345.7	345.7	345.9	343.1	344.4
BeO	580.3	580.0	580.0	579.7	579.7	579.2	581.2	580.7
CO	54.7	54.9	54.9	54.9	55.0	50.9	54.4	54.4
CO_2	144.1	145.1	145.2	145.4	145.4	143.7	144.2	144.5
SO_2	236.6	237.4	237.4	237.5	237.5	237.2	233.5	234.9
SO_3	378.7	378.8	378.8	378.3	378.4	377.1	374.7	376.7
SeO_2	291.7	292.3	292.3	292.2	292.2	291.6	288.2	290.2
TeO_2	356.2	356.5	356.3	356.0	355.9	356.1	352.0	354.2
OCF_2	213.9	214.9	214.9	215.3	215.2	213.9	213.5	214.1
NOF	97.5	97.4	97.0	96.4	96.5	91.0	95.8	96.6
NO_2F	92.0	93.5	92.9	93.9	94.5	91.0	91.5	91.6
OPF_3	241.1	241.5	241.4	241.4	241.4	244.2	240.3	240.9
SOF_2	184.1	184.5	184.8	185.1	185.1	184.4	181.3	183.1
SOF_4	260.8	261.7	261.9	262.1	262.0	263.7	258.3	258.9
SO_2F_2	169.0	169.8	170.1	170.4	170.5	171.7	168.7	168.7
BeF_2	367.0	366.6	366.9	366.7	366.6	366.6	367.3	367.2
PF_3	200.0	200.4	200.4	200.4	200.4	200.9	196.8	198.4
PF_5	377.9	378.4	378.2	378.0	377.4	379.6	377.5	377.7
AsF_3	243.1	243.3	243.3	243.4	243.4	241.7	240.1	242.4
AsF_5	431.6	431.9	431.8	431.8	431.3	431.7	429.7	430.9
SbF_3	288.1	288.2	288.3	288.3	288.2	287.6	286.1	288.0
SbF_5	487.0	487.3	487.2	487.1	486.7	487.1	486.1	486.8
SF_2	200.8	201.3	201.4	201.6	201.5	201.6	197.3	198.9
SF_4	226.9	227.5	227.8	228.2	228.1	229.2	222.8	224.9
SeF_4	297.8	298.1	298.2	298.3	298.2	297.7	293.8	296.7
TeF_4	351.2	351.4	351.4	351.4	351.3	351.8	348.7	351.4
ClF_3	246.5	246.6	247.0	247.6	247.6	248.0	247.8	245.1
ClF_5	203.5	203.6	203.2	204.7	204.3	206.1	219.3	207.8
BrF_3	293.3	293.1	293.5	293.8	293.7	293.0	292.0	292.4
BrF_5	262.8	261.6	262.0	262.6	262.4	262.3	265.3	263.5
IF_3	326.0	326.0	326.1	326.3	326.2	326.9	325.8	327.1
IF_5	278.4	282.7	282.9	282.4	283.3	280.8	283.5	283.0
F_2CCO	263.4	264.5	264.6	265.2	265.4	264.3	263.9	263.8
BMe_3	246.2	246.7	246.2	245.9	245.5	243.2	246.3	245.3
BOH_3	170.8	174.9	171.2	174.8	174.7	174.0	173.9	174.4
$AlMe_3$	360.8	361.0	360.9	360.7	360.5	359.1	360.9	359.8
$AlOH_3$	369.4	364.8	369.2	363.1	362.6	361.5	363.0	363.1
Me_3C^+	808.9	810.3	809.3	808.5	807.7	806.4	810.0	809.2
Me_3Si^+	941.5	941.9	941.7	941.4	941.5	940.2	941.9	941.2

Table S9: Directly calculated reaction energy ΔE^{FIA} in kJ/mol obtained with different methods using def2-QZVPD basis sets (expect for the composite methods) at CCSD(T*)-F12a/AVTZ-F12 structures.

LA	SVWN	BP86-D4	B97-D	BLYP-D4	PBE-D4	TPSS-D4	r²SCAN-D4
F_2	196.4	176.1	163.0	182.5	179.7	175.1	167.1
HF	222.5	187.4	177.0	181.7	189.7	187.7	194.6
H_2O	146.0	116.0	106.9	111.4	118.2	116.0	120.8
H_2CCO	208.4	156.1	137.0	136.6	160.0	160.0	174.7
H_2CO	158.8	111.6	93.3	96.7	116.1	114.0	123.7
HCO_2H	134.1	85.9	65.7	71.7	90.1	90.1	101.4
HCN	221.1	189.7	182.5	177.1	190.5	185.2	200.9
BH_3	335.9	280.3	259.8	260.0	285.3	282.5	295.7
BF_3	360.6	313.4	297.1	302.2	317.8	326.6	342.3
BF_2Cl	388.8	343.1	328.3	330.6	346.2	356.3	372.9
$BFCl_2$	406.2	361.6	346.3	347.9	363.8	374.7	391.1
BCl_3	415.6	372.1	355.9	357.5	373.6	385.3	400.7
BBr_3	443.3	399.4	386.7	384.0	400.0	410.8	429.4
AlH_3	396.4	364.0	354.0	353.3	364.6	373.8	393.0
AlF_3	485.7	450.3	441.8	444.6	453.0	459.3	483.7
AlF_2Cl	493.5	459.5	452.6	452.3	461.2	470.3	493.2
$AlFCl_2$	498.8	466.1	459.8	457.9	466.9	478.4	499.7
$AlCl_3$	502.1	470.7	464.4	461.7	470.8	484.2	503.8
GaF_3	449.6	413.8	405.5	406.8	414.9	421.6	448.2
$GaCl_3$	433.9	402.7	396.3	392.0	402.5	415.6	436.7
InF_3	429.4	396.8	389.2	389.9	398.0	403.4	428.1
$InCl_3$	413.7	387.0	379.6	377.1	386.8	398.5	419.2
H_3C^+	1171.3	1103.4	1078.7	1072.8	1110.9	1101.4	1119.0
F_3C^+	1107.0	1048.7	1032.0	1031.1	1053.4	1060.3	1086.4
H_3Si^+	1112.8	1070.5	1060.5	1050.3	1071.3	1086.5	1107.2
SiH_4	200.6	161.4	146.3	147.3	164.2	169.6	178.5
SiF_4	330.2	280.5	257.5	269.4	285.3	294.8	307.5
$SiCl_4$	349.4	303.4	282.5	288.9	305.1	318.5	328.6
F_3Si^+	1249.4	1199.9	1187.6	1185.6	1201.7	1215.7	1244.5
GeF_4	372.1	326.3	307.0	318.0	330.1	338.5	358.1
$GeCl_4$	327.4	288.1	269.1	276.2	290.2	303.0	313.0
SnF_4	422.0	380.2	368.8	372.9	382.8	390.0	415.1
$SnCl_4$	367.6	332.6	320.4	322.4	334.5	347.8	361.9
BeO	586.3	553.1	544.1	548.7	556.6	561.4	581.4
CO	101.5	68.9	55.2	61.2	72.5	70.4	71.9
CO_2	183.7	129.9	109.9	112.8	133.7	133.5	148.2
SO_2	270.8	219.5	199.6	201.6	224.0	226.9	241.8
SO_3	399.7	337.4	316.2	317.3	341.1	346.5	373.3
SeO_2	310.0	263.0	246.4	246.3	266.7	272.4	287.2
TeO_2	361.0	315.5	299.8	297.7	318.4	328.4	345.6
OCF_2	249.5	197.0	176.5	183.8	201.6	203.9	221.6
NOF	153.2	127.8	116.8	129.0	130.7	131.5	133.3
NO_2F	152.3	114.9	96.5	113.8	117.8	117.4	125.2
OPF_3	277.7	225.1	199.0	212.9	230.2	238.6	247.7
SOF_2	229.0	190.2	168.7	183.5	194.2	194.8	200.0
SOF_4	299.5	245.1	216.3	234.4	250.2	257.4	271.7
SO_2F_2	215.1	165.1	137.5	154.9	169.5	174.7	181.8
BeF_2	381.6	346.4	336.6	340.3	349.6	355.1	373.5
PF_3	239.6	194.7	172.1	183.7	200.5	204.1	210.2
PF_5	398.2	345.1	318.2	334.1	350.5	363.2	378.5
AsF_3	274.6	236.8	219.2	229.2	240.9	244.8	252.2
AsF_5	447.6	399.0	381.2	390.3	402.9	414.5	437.3
SbF_3	309.9	273.0	257.8	264.6	276.3	282.7	294.7
SbF_5	493.7	450.6	440.2	443.1	453.1	464.0	491.4
SF_2	248.7	209.8	189.1	200.7	214.0	214.9	219.1
SF_4	272.6	234.1	212.8	229.3	238.6	239.4	245.9
SeF_4	332.4	296.2	280.0	291.7	299.8	301.8	309.2
TeF_4	373.1	336.5	322.3	329.6	339.6	345.3	356.4
ClF_3	306.0	280.4	266.6	282.5	282.0	280.6	280.8
ClF_5	293.7	290.6	282.5	310.4	289.7	277.3	263.3
BrF_3	339.2	309.6	297.5	307.4	311.7	310.0	312.0
BrF_5	335.7	320.1	313.8	331.7	320.2	306.2	294.6
IF_3	359.9	332.0	319.9	327.8	334.0	332.6	334.0
IF_5	328.7	301.1	285.7	303.7	303.3	297.5	293.4
F_2CCO	305.3	253.8	235.8	237.1	258.1	261.3	279.5
BMe_3	270.0	228.1	212.1	217.2	231.9	241.6	244.8
BOH_3	202.4	160.2	143.0	148.0	163.7	170.6	180.3
$AlMe_3$	362.0	337.5	333.0	332.6	337.2	352.8	364.8
$AlOH_3$	376.7	347.7	337.8	340.6	349.8	357.2	374.7
Me_3C^+	832.5	785.3	771.5	766.2	789.7	798.9	811.5
Me_3Si^+	949.8	914.0	908.2	900.5	914.3	934.9	951.9

Table S9 continued.

LA	M06-L-D4	MN15-L	PBE0-D4	TPSSh-D4	PW6B95-D4	M06-D4	MN15
F_2	168.2	121.7	114.4	149.2	111.3	115.3	84.6
HF	178.0	164.6	192.3	188.7	185.4	179.5	188.2
H_2O	110.9	100.5	118.6	116.2	112.6	110.6	116.3
H_2CCO	155.5	170.5	176.1	166.0	163.2	159.1	176.4
H_2CO	108.2	110.5	117.1	114.3	106.5	104.9	111.4
HCO_2H	81.5	90.8	98.0	93.0	88.9	86.8	100.1
HCN	184.9	192.4	203.6	191.0	193.5	192.2	203.3
BH_3	288.3	304.3	295.3	286.8	291.6	280.2	311.1
BF_3	325.9	332.1	340.3	334.6	340.9	326.7	353.0
BF_2Cl	360.1	368.0	370.7	365.0	369.5	363.8	382.7
$BFCl_2$	380.6	390.4	389.4	383.7	386.7	386.0	401.1
BCl_3	392.2	404.2	400.1	394.4	395.9	398.9	412.0
BBr_3	411.3	431.1	428.2	420.6	422.3	426.9	440.4
AlH_3	385.4	402.2	384.2	380.6	382.9	375.1	401.0
AlF_3	481.7	477.5	477.1	468.0	478.5	478.8	486.7
AlF_2Cl	491.0	490.5	486.3	479.2	486.8	485.7	498.1
$AlFCl_2$	497.1	499.8	492.7	487.2	492.5	490.1	505.9
$AlCl_3$	501.2	506.6	497.0	493.0	496.2	492.7	511.2
GaF_3	440.4	433.9	441.0	431.1	440.7	442.4	452.5
$GaCl_3$	429.0	436.2	428.3	424.2	423.9	419.8	442.5
InF_3	424.4	409.6	422.3	412.4	421.6	425.8	428.0
$InCl_3$	418.8	411.9	409.6	406.2	405.3	407.1	416.9
H_3C^+	1122.0	1103.7	1119.8	1105.8	1109.1	1104.4	1119.7
F_3C^+	1061.4	1073.5	1091.9	1074.9	1086.0	1075.3	1099.6
H_3Si^+	1107.1	1114.5	1096.8	1094.9	1094.4	1091.4	1116.2
SiH_4	171.9	193.4	176.4	173.9	176.0	164.0	190.8
SiF_4	299.7	299.3	303.4	300.8	308.2	293.0	319.4
$SiCl_4$	322.8	331.6	323.5	324.1	324.6	319.4	340.6
F_3Si^+	1237.4	1233.2	1241.9	1230.0	1242.9	1238.1	1253.1
GeF_4	349.2	347.0	349.0	345.0	351.2	347.8	363.2
$GeCl_4$	307.2	319.8	304.4	306.9	303.6	295.9	320.6
SnF_4	408.8	400.5	404.1	397.5	404.7	410.5	414.7
$SnCl_4$	360.9	364.2	350.1	352.3	348.5	347.4	362.8
BeO	570.3	575.6	583.3	571.6	580.3	572.9	585.3
CO	64.2	56.0	62.9	66.4	55.6	55.8	57.5
CO_2	118.7	136.8	150.8	140.1	140.2	127.9	155.1
SO_2	223.3	234.1	243.8	234.2	233.4	228.1	240.5
SO_3	348.9	369.4	383.3	362.6	374.9	367.5	389.9
SeO_2	273.7	286.2	294.5	282.6	285.8	285.1	296.4
TeO_2	331.5	341.0	354.4	341.5	347.2	344.0	356.8
OCF_2	193.9	205.5	217.5	209.8	209.8	201.7	220.8
NOF	128.7	103.7	108.6	122.3	105.2	104.6	96.4
NO_2F	103.8	88.7	101.1	110.4	95.9	88.3	94.3
OPF_3	235.8	235.3	245.8	243.7	247.3	230.9	256.2
SOF_2	183.2	168.8	191.9	193.6	187.6	181.0	184.0
SOF_4	253.3	253.9	265.1	262.5	264.1	254.8	271.5
SO_2F_2	160.5	157.6	176.0	176.4	173.5	160.7	179.0
BeF_2	366.4	364.5	369.0	362.1	369.5	363.7	377.4
PF_3	201.2	194.0	206.1	205.8	205.2	193.5	206.9
PF_5	372.7	373.9	375.2	371.7	380.7	367.0	390.7
AsF_3	245.1	238.5	247.5	246.9	246.2	243.0	250.4
AsF_5	425.3	429.8	430.5	424.2	433.1	428.3	446.7
SbF_3	288.6	282.4	289.4	287.0	288.2	287.4	294.4
SbF_5	483.4	479.1	483.4	474.8	484.6	488.1	495.3
SF_2	205.1	190.2	209.1	212.6	204.2	195.8	197.6
SF_4	226.3	209.6	233.1	236.9	229.2	221.6	220.5
SeF_4	297.8	282.5	301.5	302.1	299.2	296.6	296.2
TeF_4	348.5	341.1	351.6	349.3	350.1	349.5	355.8
ClF_3	262.4	230.8	253.4	269.0	248.4	243.1	226.3
ClF_5	240.7	158.4	206.3	244.3	202.9	199.8	153.9
BrF_3	304.2	272.1	297.1	304.0	292.8	295.4	279.3
BrF_5	284.1	201.3	263.1	284.1	259.9	263.2	222.7
IF_3	330.5	312.9	326.7	329.5	322.9	329.2	326.2
IF_5	293.5	265.2	283.7	289.8	284.4	290.0	288.1
F_2CCO	258.0	272.4	273.9	267.0	264.2	259.8	273.5
BMe_3	236.4	253.9	244.0	245.2	244.3	236.3	259.3
BOH_3	167.7	175.0	177.7	175.4	176.5	168.5	190.3
$AlMe_3$	364.3	373.2	356.2	358.7	357.7	355.9	371.6
$AlOH_3$	374.4	372.0	368.1	363.5	368.4	366.9	375.6
Me_3C^+	810.6	812.4	818.3	809.0	810.3	813.8	827.6
Me_3Si^+	950.0	955.2	946.2	945.3	945.4	942.8	961.5

Table S9 continued.

LA	M06-2X-D4	B3LYP-D4	BHLYP-D4	ωB97M-V	ωB97X	ωB97X-D	ωB97X-V	CAM-B3LYP-D4
F_2	56.2	131.8	61.0	81.0	85.4	92.3	79.9	102.2
HF	197.6	186.8	189.9	184.2	187.9	182.5	186.8	193.5
H_2O	121.2	114.1	115.1	112.5	115.8	111.4	113.3	118.4
H_2CCO	178.4	153.3	170.1	164.4	165.3	160.1	167.9	163.8
H_2CO	113.5	100.9	102.6	103.4	104.5	100.3	105.1	105.4
HCO_2H	103.8	81.2	89.9	91.1	89.0	81.7	92.5	89.9
HCN	212.3	190.4	203.3	199.9	196.8	197.1	196.9	196.9
BH_3	293.3	272.7	282.2	283.4	277.5	277.2	279.6	281.0
BF_3	360.3	324.3	348.5	338.1	335.5	326.5	339.7	338.7
BF_2Cl	391.7	354.0	379.9	368.3	364.0	358.5	368.2	366.7
$BFCl_2$	410.0	371.9	398.7	387.6	383.0	378.9	386.9	384.3
BCl_3	419.7	381.9	409.3	399.5	395.0	391.4	398.9	394.6
BBr_3	448.2	409.8	439.1	428.9	421.2	417.9	427.4	422.9
AlH_3	398.2	371.8	392.6	379.5	382.5	377.9	380.2	379.9
AlF_3	504.8	467.7	494.3	476.0	478.4	470.4	477.4	479.4
AlF_2Cl	512.3	476.0	503.2	485.8	487.7	480.2	487.2	487.6
$AlFCl_2$	517.1	481.9	509.6	492.8	494.2	486.9	494.3	493.3
$AlCl_3$	520.1	485.8	514.0	497.7	498.8	491.3	499.3	497.2
GaF_3	472.4	431.6	459.9	440.9	445.5	438.0	442.5	444.1
$GaCl_3$	456.5	416.0	444.4	429.4	430.9	423.3	432.4	428.2
InF_3	449.7	413.3	440.1	425.3	430.3	421.7	426.2	426.4
$InCl_3$	436.3	398.8	424.9	413.0	418.5	409.2	416.7	411.6
H_3C^+	1111.1	1086.5	1092.0	1093.8	1094.5	1096.1	1095.9	1096.7
F_3C^+	1115.6	1067.0	1106.6	1082.1	1079.5	1073.3	1086.6	1086.2
H_3Si^+	1104.0	1075.4	1101.9	1085.7	1086.9	1086.8	1087.7	1086.5
SiH_4	180.2	159.9	172.6	172.2	167.4	163.8	166.9	165.3
SiF_4	322.7	288.3	309.0	303.9	299.7	289.7	303.1	302.2
$SiCl_4$	345.2	307.2	327.2	325.4	316.8	311.7	321.1	316.8
F_3Si^+	1269.5	1222.8	1265.0	1233.2	1233.8	1229.7	1236.1	1239.5
GeF_4	373.0	337.1	359.2	350.6	346.9	337.4	349.3	349.7
$GeCl_4$	330.2	290.4	306.7	305.0	295.8	290.8	302.2	297.0
SnF_4	430.7	393.9	418.6	406.2	406.7	397.3	405.7	406.2
$SnCl_4$	373.8	337.7	356.5	351.9	347.8	339.4	351.3	345.5
BeO	602.1	574.2	603.4	578.2	577.7	570.4	581.7	587.0
CO	51.8	56.1	48.6	50.8	54.1	51.0	51.8	54.8
CO_2	159.0	130.7	149.3	143.4	142.4	136.0	146.4	144.4
SO_2	248.1	221.7	244.8	232.6	234.4	227.1	236.7	233.9
SO_3	401.0	356.3	401.9	379.9	378.8	369.6	383.2	380.0
SeO_2	311.5	272.4	305.1	289.4	290.5	282.1	293.5	288.5
TeO_2	385.9	330.6	372.7	355.3	356.7	345.6	359.6	351.6
OCF_2	229.8	200.2	217.1	212.9	210.3	200.6	215.5	213.6
NOF	91.8	112.9	88.1	93.9	94.7	94.4	92.9	102.7
NO_2F	94.6	102.3	81.7	91.3	87.9	84.2	90.8	98.9
OPF_3	262.7	229.2	246.3	244.7	239.2	229.3	243.6	242.6
SOF_2	195.6	184.3	182.0	185.4	181.7	175.8	183.2	186.6
SOF_4	283.1	249.6	264.8	263.8	256.6	246.5	263.2	262.2
SO_2F_2	189.2	163.1	169.6	173.3	166.8	157.3	172.2	172.5
BeF_2	390.5	359.5	381.2	365.6	368.1	360.4	367.4	370.1
PF_3	215.0	191.9	198.7	202.5	199.3	191.1	200.2	199.3
PF_5	398.9	357.7	384.1	375.2	368.9	359.1	374.4	372.8
AsF_3	257.4	237.6	245.7	244.3	241.5	234.4	242.9	243.3
AsF_5	457.6	416.0	446.0	431.4	424.5	415.2	430.9	430.5
SbF_3	305.3	278.3	293.9	288.5	287.4	279.4	287.8	286.5
SbF_5	516.0	471.0	504.6	486.1	483.6	474.3	486.0	485.6
SF_2	207.2	199.9	195.1	199.8	198.0	193.3	198.0	201.1
SF_4	236.6	227.2	220.3	226.5	220.9	214.7	224.2	227.7
SeF_4	306.3	295.5	296.6	295.1	291.5	286.1	293.8	297.4
TeF_4	364.5	342.2	355.1	348.0	346.0	339.3	348.1	348.5
ClF_3	233.4	261.3	229.0	233.6	229.0	234.0	229.9	245.6
ClF_5	164.2	242.6	144.9	172.9	164.1	181.6	163.4	195.8
BrF_3	279.2	298.3	282.1	279.9	281.2	281.1	279.3	290.1
BrF_5	208.7	287.1	219.5	234.4	236.4	243.7	229.8	256.2
IF_3	319.9	324.8	317.4	313.4	317.8	315.2	315.0	320.9
IF_5	267.2	290.1	268.6	271.9	273.1	270.8	269.6	281.3
F_2CCO	280.4	253.7	271.1	264.6	262.5	256.5	267.9	264.4
BMe_3	257.0	228.7	240.2	239.8	229.2	228.8	235.8	234.0
BOH_3	194.9	162.1	177.0	178.1	173.0	164.5	177.9	172.6
$AlMe_3$	376.3	349.4	369.7	355.2	352.3	348.7	354.8	355.2
$AlOH_3$	389.5	358.0	378.1	367.7	367.9	361.0	367.8	365.8
Me_3C^+	835.9	792.1	818.2	813.1	812.6	806.8	817.9	808.1
Me_3Si^+	964.9	929.2	962.0	943.4	941.2	939.5	946.1	942.6

Table S9 continued.

LA	LH07s-SVWN-D4	LH07t-SVWN-D4	LH12ct-ssirPW92-D4	LH12ct-ssifPW92-D4	LH14t-calPBE-D4
F_2	112.3	111.6	85.9	76.2	117.2
HF	182.0	184.1	178.7	177.6	188.0
H_2O	109.0	110.4	106.0	105.1	114.3
H_2CCO	157.2	159.6	160.3	161.9	165.7
H_2CO	101.8	100.5	96.2	95.9	106.7
HCO_2H	79.6	80.8	78.4	78.6	87.9
HCN	195.1	193.2	195.2	197.3	195.0
BH_3	273.5	272.7	269.8	270.4	275.9
BF_3	315.7	324.7	322.4	322.0	332.5
BF_2Cl	349.1	357.2	356.6	356.7	363.9
$BFCl_2$	370.4	377.6	378.7	379.3	383.2
BCl_3	383.4	389.6	392.3	393.5	394.3
BBr_3	410.7	416.3	418.1	419.0	421.2
AlH_3	370.8	376.9	379.2	379.8	378.3
AlF_3	453.8	466.1	464.3	463.6	472.6
AlF_2Cl	465.6	476.4	475.1	474.5	482.4
$AlFCl_2$	474.9	484.3	484.0	483.7	489.3
$AlCl_3$	482.1	490.2	491.2	491.3	494.1
GaF_3	425.5	434.9	436.5	436.8	438.3
$GaCl_3$	417.3	421.8	424.3	424.9	424.6
InF_3	407.1	415.7	416.1	416.2	420.8
$InCl_3$	396.9	401.8	402.3	402.3	406.4
H_3C^+	1092.6	1088.5	1081.7	1082.1	1093.1
F_3C^+	1063.8	1071.6	1072.3	1073.7	1081.4
H_3Si^+	1075.5	1080.9	1079.3	1079.0	1086.1
SiH_4	160.2	158.3	159.9	161.0	161.4
SiF_4	273.6	283.0	277.9	276.6	292.3
$SiCl_4$	301.5	308.9	307.6	307.3	316.9
F_3Si^+	1215.8	1226.1	1227.2	1227.7	1232.3
GeF_4	322.2	332.7	329.0	327.8	341.3
$GeCl_4$	285.0	290.1	289.5	289.0	297.3
SnF_4	382.2	392.8	391.4	390.8	399.4
$SnCl_4$	332.9	338.1	338.1	337.6	343.5
BeO	560.5	571.7	570.1	569.7	578.9
CO	51.2	51.5	45.0	43.4	57.6
CO_2	132.4	135.2	134.0	134.9	142.8
SO_2	225.1	229.2	231.3	233.1	235.2
SO_3	361.7	367.7	374.2	377.6	372.5
SeO_2	277.3	282.0	286.8	289.3	287.9
TeO_2	337.7	343.4	350.9	354.2	348.8
OCF_2	196.9	202.8	201.2	201.5	210.6
NOF	99.2	100.2	88.1	83.6	106.4
NO_2F	89.3	93.1	82.5	78.8	100.8
OPF_3	219.9	226.3	223.7	223.2	234.2
SOF_2	176.0	179.3	174.9	173.8	186.4
SOF_4	240.7	248.9	247.3	247.1	256.8
SO_2F_2	154.2	159.4	155.8	155.0	167.5
BeF_2	346.0	356.7	353.5	352.4	363.3
PF_3	183.5	188.4	184.9	184.3	196.2
PF_5	347.8	358.2	358.4	358.6	366.0
AsF_3	228.0	234.2	231.3	230.5	241.5
AsF_5	406.3	417.1	418.7	419.1	424.0
SbF_3	271.2	278.3	277.8	277.6	284.6
SbF_5	463.2	474.1	477.5	478.4	479.4
SF_2	192.9	196.0	190.0	188.5	203.4
SF_4	217.0	222.5	217.8	216.3	229.5
SeF_4	285.7	292.0	289.0	287.9	298.2
TeF_4	336.1	343.1	343.4	343.4	348.2
ClF_3	249.0	251.9	240.0	235.4	258.3
ClF_5	222.6	222.9	201.0	191.9	227.2
BrF_3	289.4	293.2	285.7	283.0	298.7
BrF_5	270.7	273.5	258.1	252.0	276.7
IF_3	319.8	324.1	320.2	318.8	328.2
IF_5	276.3	281.5	274.1	271.2	285.9
F_2CCO	255.6	257.5	256.2	256.7	264.3
BMe_3	229.5	235.0	237.5	238.3	237.3
BOH_3	157.0	161.2	160.1	160.1	167.3
$AlMe_3$	345.1	351.8	353.6	353.4	353.3
$AlOH_3$	348.3	355.4	354.7	354.3	360.1
Me_3C^+	796.4	804.5	808.3	809.9	810.6
Me_3Si^+	926.6	933.6	934.0	933.6	938.8

Table S9 continued.

LA	LH20t-D4	LHJ14	LHJ-HF	LHJ-HFcal-D4	mPSTS-noa2	TMHF-D4	TMHF-3P
F_2	95.8	152.0	104.9	99.5	135.1	127.4	118.2
HF	186.3	194.4	182.5	183.7	181.6	176.7	176.6
H_2O	112.8	120.9	108.2	108.9	108.9	105.8	105.1
H_2CCO	167.9	174.5	159.1	165.0	154.1	136.1	136.8
H_2CO	105.3	122.0	103.5	107.3	102.8	91.2	89.5
HCO_2H	89.0	104.6	86.2	89.6	80.4	71.4	69.6
HCN	193.2	191.4	184.0	187.4	186.9	178.7	178.7
BH_3	276.4	291.4	279.4	283.7	276.1	277.4	275.3
BF_3	341.2	347.1	341.1	341.0	323.1	331.7	329.8
BF_2Cl	372.5	372.5	366.3	367.0	354.2	354.6	353.0
$BFCl_2$	391.3	388.7	382.7	384.1	372.8	368.7	367.4
BCl_3	402.0	398.3	392.7	394.7	382.9	376.7	375.5
BBr_3	427.4	425.3	419.9	421.9	409.5	401.5	400.8
AlH_3	382.2	382.0	377.7	379.2	371.3	376.7	374.7
AlF_3	479.5	481.3	475.2	472.7	458.2	475.6	473.5
AlF_2Cl	489.4	488.6	483.8	482.1	469.8	482.6	480.6
$AlFCl_2$	496.0	493.7	489.9	488.8	477.7	487.2	485.3
$AlCl_3$	501.0	497.3	494.4	493.7	482.8	490.4	488.5
GaF_3	445.0	442.6	437.5	436.4	421.7	434.0	432.9
$GaCl_3$	429.5	426.6	422.7	423.4	414.1	416.9	415.1
InF_3	428.3	424.0	420.1	418.8	404.9	417.8	417.4
$InCl_3$	411.8	409.2	406.3	406.6	397.3	404.0	402.9
H_3C^+	1088.8	1109.6	1091.2	1099.4	1096.0	1082.2	1082.2
F_3C^+	1092.2	1086.9	1081.1	1084.7	1065.1	1058.8	1060.0
H_3Si^+	1091.2	1092.5	1087.7	1090.2	1088.5	1079.9	1079.0
SiH_4	165.3	177.2	167.0	169.9	161.8	165.7	162.4
SiF_4	300.9	317.2	308.0	306.1	286.5	304.0	299.4
$SiCl_4$	326.0	331.9	319.6	319.1	311.5	310.7	306.7
F_3Si^+	1241.5	1238.4	1236.3	1236.8	1220.5	1226.8	1226.4
GeF_4	347.3	361.8	349.2	347.0	331.2	349.2	344.9
$GeCl_4$	302.2	312.5	297.2	296.6	294.0	294.2	289.6
SnF_4	405.5	413.8	402.7	400.5	384.9	402.9	399.9
$SnCl_4$	347.6	358.3	345.6	344.8	339.0	345.9	342.0
BeO	585.3	578.0	577.0	575.9	564.8	574.9	575.9
CO	54.2	71.6	51.2	51.9	56.5	47.4	45.1
CO_2	145.9	153.1	139.0	143.2	129.5	114.9	115.7
SO_2	240.5	244.0	236.3	240.9	222.2	216.8	218.1
SO_3	382.6	379.1	380.3	385.6	349.6	352.7	355.4
SeO_2	295.9	292.7	292.0	295.8	271.9	274.0	275.5
TeO_2	359.8	351.5	357.2	361.0	331.2	340.0	341.9
OCF_2	214.7	224.9	210.4	212.8	196.8	194.1	192.8
NOF	98.5	126.1	96.6	93.8	110.5	105.3	99.6
NO_2F	94.3	123.1	88.8	86.6	95.9	89.2	83.4
OPF_3	242.3	258.7	246.3	246.8	227.5	238.4	234.5
SOF_2	185.4	205.5	182.9	182.7	179.9	181.1	177.5
SOF_4	262.9	280.5	262.8	263.6	244.8	256.0	251.8
SO_2F_2	170.6	191.7	170.2	170.9	159.8	163.7	159.5
BeF_2	368.2	373.2	366.5	364.5	353.2	365.3	363.6
PF_3	200.9	217.8	202.4	202.9	191.7	198.5	195.3
PF_5	379.6	387.2	380.9	381.2	355.1	375.6	371.3
AsF_3	244.2	258.8	243.0	242.0	234.1	241.7	238.8
AsF_5	433.4	440.5	431.6	431.2	408.8	428.8	425.0
SbF_3	288.9	298.4	286.5	285.6	275.1	285.0	282.6
SbF_5	488.6	490.2	482.8	481.9	460.6	481.4	478.7
SF_2	202.5	221.0	199.5	199.6	200.7	197.5	194.3
SF_4	227.7	250.1	223.8	223.1	221.7	224.8	220.2
SeF_4	298.6	315.3	294.4	293.2	287.8	293.8	290.5
TeF_4	351.8	360.9	347.5	346.7	335.9	345.1	342.7
ClF_3	247.7	276.8	239.6	235.6	255.9	246.6	240.4
ClF_5	200.3	255.3	182.7	173.1	227.3	211.4	198.5
BrF_3	294.2	314.1	287.4	285.1	291.7	288.5	285.3
BrF_5	260.0	300.9	245.3	239.2	267.2	256.7	250.5
IF_3	326.8	339.6	320.5	318.8	318.2	318.9	317.2
IF_5	281.8	306.9	276.4	272.4	274.5	280.6	276.3
F_2CCO	267.0	277.4	262.3	266.0	254.5	242.4	241.0
BMe_3	245.2	246.9	232.5	236.1	227.3	221.8	218.3
BOH_3	172.4	186.0	173.4	174.6	162.7	164.2	161.0
$AlMe_3$	359.5	356.3	349.0	349.5	344.9	348.9	345.1
$AlOH_3$	364.4	371.4	362.2	361.5	352.9	362.5	359.5
Me_3C^+	820.3	816.3	806.6	813.0	794.6	782.5	783.0
Me_3Si^+	947.9	944.8	941.7	944.0	933.4	928.3	927.1

Table S9 continued.

LA	ωLH22t-D4	scLH22t-D4	scLH22ta-D4	ωLH23tdE-D4
F_2	80.1	113.4	159.8	88.3
HF	187.0	186.2	183.0	186.9
H_2O	112.8	112.6	109.7	112.7
H_2CCO	166.9	167.2	152.6	166.6
H_2CO	103.7	105.0	100.9	103.6
HCO_2H	89.6	88.7	78.3	89.6
HCN	195.2	193.1	178.5	195.1
BH_3	282.9	276.3	263.7	283.0
BF_3	343.4	341.1	329.6	343.4
BF_2Cl	372.9	372.2	355.4	372.9
$BFCl_2$	391.4	391.0	371.1	391.4
BCl_3	402.5	401.7	379.8	402.5
BBr_3	428.5	427.0	400.8	428.4
AlH_3	383.8	380.8	367.3	382.8
AlF_3	480.9	479.4	467.0	480.9
AlF_2Cl	490.4	489.3	474.7	490.4
$AlFCl_2$	496.8	495.9	479.8	496.8
$AlCl_3$	501.4	500.8	483.6	501.4
GaF_3	446.0	444.9	433.3	446.0
$GaCl_3$	430.8	429.3	413.3	430.8
InF_3	429.2	427.4	416.6	428.5
$InCl_3$	413.7	410.2	396.1	412.1
H_3C^+	1095.3	1088.2	1073.8	1095.2
F_3C^+	1091.6	1092.2	1069.8	1091.6
H_3Si^+	1091.7	1088.2	1071.6	1089.2
SiH_4	167.5	164.8	154.9	167.1
SiF_4	305.6	300.9	292.8	305.6
$SiCl_4$	326.0	325.8	307.9	326.0
F_3Si^+	1243.0	1241.4	1220.6	1243.0
GeF_4	351.1	347.3	337.2	351.1
$GeCl_4$	302.5	302.1	285.8	302.5
SnF_4	407.8	404.5	393.5	407.1
$SnCl_4$	349.0	345.8	331.3	347.5
BeO	584.9	584.7	571.0	584.9
CO	51.1	54.4	55.1	51.1
CO_2	146.6	145.9	130.4	146.6
SO_2	238.7	239.2	224.7	238.6
SO_3	385.4	381.6	356.8	385.1
SeO_2	295.5	291.2	270.9	293.9
TeO_2	361.4	349.9	325.1	354.6
OCF_2	215.8	214.6	202.6	215.8
NOF	91.6	100.1	114.3	91.7
NO_2F	90.4	94.0	101.8	90.1
OPF_3	245.6	242.3	235.0	245.6
SOF_2	183.9	185.4	183.4	183.9
SOF_4	265.8	262.8	253.6	265.8
SO_2F_2	172.8	170.6	163.7	172.8
BeF_2	370.4	368.3	357.1	370.4
PF_3	201.9	200.9	198.2	201.9
PF_5	381.7	379.5	369.7	381.7
AsF_3	244.1	244.1	238.3	244.1
AsF_5	435.8	433.4	419.0	435.8
SbF_3	289.2	286.2	276.9	286.2
SbF_5	490.1	487.1	471.5	489.1
SF_2	199.9	202.6	203.3	199.9
SF_4	225.7	227.8	227.3	225.7
SeF_4	295.8	298.5	294.7	295.8
TeF_4	350.3	350.5	340.9	348.5
ClF_3	234.8	248.9	261.3	234.8
ClF_5	173.9	204.2	244.7	173.9
BrF_3	283.7	294.4	300.1	283.6
BrF_5	238.8	261.2	287.9	238.8
IF_3	318.4	326.4	328.0	317.8
IF_5	273.5	282.6	292.2	275.0
F_2CCO	266.3	268.6	255.8	267.7
BMe_3	243.1	243.7	231.0	242.2
BOH_3	175.5	172.2	161.0	175.5
$AlMe_3$	357.5	358.5	345.0	356.9
$AlOH_3$	365.9	364.3	351.9	365.9
Me_3C^+	819.7	816.5	800.4	816.9
Me_3Si^+	947.3	945.8	928.5	945.8

Table S9 continued.

LA	B2PLYP-D4	B2GP-PLYP-D4	B2K-PLYP	B2T-PLYP	PWPB95-D4
F_2	135.3	117.4	106.2	121.5	123.2
HF	185.1	186.1	186.3	185.3	183.7
H_2O	113.6	114.1	114.1	113.2	112.1
H_2CCO	153.2	157.5	158.9	154.3	162.2
H_2CO	98.1	98.9	98.6	97.2	102.5
HCO_2H	81.2	83.8	84.4	81.2	87.7
HCN	189.5	192.6	193.7	190.7	192.1
BH_3	272.6	275.7	276.6	273.3	288.4
BF_3	329.8	336.0	338.6	332.8	341.3
BF_2Cl	359.1	365.6	368.3	361.9	370.3
$BFCl_2$	376.9	383.6	386.2	379.4	387.9
BCl_3	386.9	393.7	396.1	389.0	397.7
BBr_3	413.9	421.0	423.4	416.3	424.2
AlH_3	375.2	380.1	381.7	376.5	383.5
AlF_3	472.4	478.3	480.8	475.3	479.0
AlF_2Cl	481.5	487.8	490.3	484.3	488.0
$AlFCl_2$	488.1	494.6	497.0	490.6	494.3
$AlCl_3$	492.1	498.8	501.0	494.2	498.2
GaF_3	437.6	444.2	446.8	440.5	442.2
$GaCl_3$	423.7	430.6	432.8	425.7	428.0
InF_3	422.1	429.1	432.1	425.4	424.3
$InCl_3$	407.5	414.0	416.2	409.5	409.6
H_3C^+	1086.1	1089.4	1090.7	1087.3	1103.5
F_3C^+	1072.9	1082.4	1087.0	1078.5	1086.9
H_3Si^+	1081.9	1088.8	1091.9	1085.4	1094.4
SiH_4	160.6	163.9	164.5	160.4	174.2
SiF_4	292.3	297.4	299.2	294.2	306.4
$SiCl_4$	312.0	317.4	319.0	313.2	324.9
F_3Si^+	1229.6	1239.1	1243.3	1234.8	1242.8
GeF_4	341.8	347.2	349.1	343.7	351.0
$GeCl_4$	293.9	298.0	298.7	293.9	304.0
SnF_4	399.9	406.0	408.3	402.2	405.5
$SnCl_4$	342.4	346.9	347.8	342.5	349.6
BeO	572.7	579.1	582.0	576.9	575.8
CO	49.0	46.5	44.5	46.4	50.0
CO_2	125.9	129.7	131.1	127.6	135.9
SO_2	216.9	221.6	223.2	218.8	226.8
SO_3	357.6	368.0	372.5	363.0	371.6
SeO_2	261.8	268.1	270.6	265.1	274.4
TeO_2	318.4	326.8	330.5	323.2	333.9
OCF_2	201.3	205.6	207.1	202.7	208.6
NOF	109.0	103.5	99.6	103.6	102.8
NO_2F	97.7	93.4	90.0	92.7	93.2
OPF_3	230.8	235.0	236.1	231.4	244.5
SOF_2	180.3	179.7	178.3	177.9	183.6
SOF_4	251.3	255.3	256.2	251.4	262.9
SO_2F_2	161.2	162.7	162.3	159.8	170.5
BeF_2	362.9	367.8	369.9	365.5	369.5
PF_3	192.1	194.1	194.2	191.6	201.7
PF_5	363.6	370.2	372.6	365.6	379.7
AsF_3	237.7	239.7	239.8	237.3	243.5
AsF_5	422.8	430.3	433.0	425.4	433.8
SbF_3	281.7	285.6	286.7	282.4	287.5
SbF_5	479.0	487.2	490.3	482.1	486.4
SF_2	197.6	196.8	195.6	195.5	200.6
SF_4	223.7	222.4	220.6	220.5	225.9
SeF_4	292.6	292.8	291.6	290.5	295.4
TeF_4	344.2	347.5	348.0	344.0	348.6
ClF_3	260.1	253.5	248.9	253.3	250.0
ClF_5	242.4	223.2	211.3	224.8	211.1
BrF_3	295.6	292.1	289.0	291.2	290.4
BrF_5	277.6	263.0	253.4	264.2	255.3
IF_3	323.3	322.0	320.2	320.3	320.1
IF_5	283.9	279.1	275.2	277.9	277.6
F_2CCO	254.0	258.0	259.1	254.9	262.0
BMe_3	233.3	237.2	237.2	231.5	247.0
BOH_3	168.9	173.6	175.2	169.7	179.5
$AlMe_3$	354.3	359.0	359.5	353.6	360.4
$AlOH_3$	363.6	368.6	370.1	364.7	370.2
Me_3C^+	805.4	814.1	817.3	807.5	819.7
Me_3Si^+	940.0	948.5	951.4	942.5	950.7

Table S9 continued.

LA	PBE0-DH-D3(BJ)	DSD-BLYP-D3(BJ)	DSD-PBEP86-D3(BJ)	DSD-PBEB95-D3(BJ)
F_2	93.8	120.5	119.2	112.6
HF	192.0	184.4	185.6	182.5
H_2O	118.2	113.0	114.6	111.2
H_2CCO	179.9	154.4	161.6	160.0
H_2CO	114.9	96.0	100.7	98.4
HCO_2H	99.2	81.2	86.7	84.8
HCN	206.7	190.5	194.8	191.8
BH_3	296.7	273.3	280.5	284.2
BF_3	349.5	333.8	337.7	340.9
BF_2Cl	380.8	363.4	368.0	370.2
$BFCl_2$	400.0	381.3	386.8	388.3
BCl_3	410.9	391.4	397.6	398.5
BBr_3	439.4	418.4	424.9	425.1
AlH_3	392.3	378.1	382.4	383.0
AlF_3	488.2	476.6	478.4	480.3
AlF_2Cl	498.3	486.3	488.5	489.8
$AlFCl_2$	505.8	493.2	495.8	496.6
$AlCl_3$	510.7	497.4	500.4	500.9
GaF_3	453.3	442.5	444.5	444.6
$GaCl_3$	442.3	429.4	433.2	431.8
InF_3	436.3	428.7	430.2	429.0
$InCl_3$	423.4	413.3	416.4	414.5
H_3C^+	1119.8	1086.9	1095.5	1099.2
F_3C^+	1106.0	1079.0	1084.5	1087.6
H_3Si^+	1108.0	1086.5	1091.6	1093.7
SiH_4	180.0	161.7	167.8	170.6
SiF_4	311.6	295.4	299.0	304.2
$SiCl_4$	333.4	316.3	321.8	324.1
F_3Si^+	1258.1	1235.4	1238.8	1243.3
GeF_4	358.5	345.4	348.0	350.4
$GeCl_4$	313.2	297.1	302.1	303.2
SnF_4	416.2	404.8	406.7	407.2
$SnCl_4$	361.5	346.4	350.4	350.7
BeO	590.7	575.7	576.6	577.5
CO	56.7	44.3	46.8	44.7
CO_2	153.4	125.9	131.8	132.1
SO_2	248.3	217.4	223.2	224.1
SO_3	398.1	363.1	369.1	371.5
SeO_2	300.0	263.1	268.7	271.8
TeO_2	362.9	321.8	327.9	332.7
OCF_2	221.9	202.5	207.1	206.4
NOF	98.7	103.1	102.0	96.8
NO_2F	91.9	92.5	92.7	87.0
OPF_3	251.4	232.5	237.0	241.2
SOF_2	189.0	177.9	180.3	179.0
SOF_4	270.3	253.0	257.3	259.6
SO_2F_2	176.7	160.4	164.4	165.6
BeF_2	377.1	365.9	368.0	369.7
PF_3	207.7	192.4	196.5	198.6
PF_5	385.8	367.9	372.2	378.1
AsF_3	250.3	238.2	240.9	241.3
AsF_5	443.5	428.1	431.3	434.0
SbF_3	297.1	284.6	287.5	287.6
SbF_5	499.3	485.6	487.9	488.8
SF_2	206.0	195.5	198.4	196.8
SF_4	229.0	221.1	223.2	221.1
SeF_4	300.8	291.1	292.7	291.6
TeF_4	357.9	346.3	348.5	347.9
ClF_3	242.9	254.5	253.6	245.1
ClF_5	176.2	230.1	223.9	203.1
BrF_3	291.2	291.6	291.4	286.3
BrF_5	238.2	265.3	259.5	245.6
IF_3	325.0	321.6	321.9	318.2
IF_5	275.2	278.7	276.1	272.0
F_2CCO	278.3	254.5	260.0	259.1
BMe_3	248.5	235.2	241.8	243.9
BOH_3	184.6	172.4	178.1	178.8
$AlMe_3$	365.2	357.1	360.0	360.3
$AlOH_3$	377.6	367.5	370.7	371.2
Me_3C^+	831.0	813.1	822.4	822.3
Me_3Si^+	959.1	945.2	950.0	951.3

Table S9 continued.

LA	$Pr^2SCAN50-D4$	$Pr^2SCAN69-D4$	$\varkappa Pr^2SCAN50-D4$	$\omega Pr^2SCAN50-D4$	ωB97M(2)
F_2	127.3	110.7	112.1	114.6	103.2
HF	190.3	187.5	190.4	188.7	181.4
H_2O	117.1	114.9	117.2	115.9	111.8
H_2CCO	172.7	168.1	171.3	167.4	160.5
H_2CO	110.4	103.3	110.5	105.9	98.7
HCO_2H	95.7	91.3	95.8	92.1	86.5
HCN	201.2	198.2	199.2	197.8	193.5
BH_3	291.5	286.5	289.8	287.0	281.3
BF_3	348.2	347.1	347.0	342.5	337.2
BF_2Cl	379.1	377.7	377.4	372.3	365.6
$BFCl_2$	397.8	396.5	396.1	391.0	384.2
BCl_3	408.2	407.2	406.7	402.0	396.1
BBr_3	436.4	434.7	434.8	429.7	424.4
AlH_3	393.9	391.6	392.6	386.5	379.9
AlF_3	489.1	488.2	487.8	481.2	477.8
AlF_2Cl	499.1	498.3	497.9	491.2	487.2
$AlFCl_2$	506.2	505.6	505.2	498.5	494.1
$AlCl_3$	510.6	510.1	509.8	503.2	498.7
GaF_3	455.4	455.1	454.3	447.5	442.2
$GaCl_3$	443.4	443.1	442.9	436.1	433.0
InF_3	437.0	438.2	436.7	431.0	429.7
$InCl_3$	424.8	424.9	424.9	418.8	419.3
H_3C^+	1110.5	1103.3	1109.3	1104.8	1096.3
F_3C^+	1099.7	1099.5	1096.7	1090.7	1081.3
H_3Si^+	1107.8	1104.3	1106.3	1097.9	1091.0
SiH_4	177.6	174.5	176.1	172.9	166.4
SiF_4	309.7	307.2	308.5	304.5	299.3
$SiCl_4$	331.3	329.1	329.5	325.5	319.1
F_3Si^+	1256.1	1255.0	1252.9	1244.6	1237.6
GeF_4	359.3	357.2	358.3	352.8	350.6
$GeCl_4$	311.4	308.4	310.1	304.7	302.0
SnF_4	417.4	415.9	416.7	410.2	409.1
$SnCl_4$	360.1	357.4	359.5	353.4	354.2
BeO	586.4	585.8	587.2	580.5	575.8
CO	53.8	46.3	52.8	50.3	45.0
CO_2	144.9	139.6	143.0	140.2	131.9
SO_2	237.3	231.6	237.8	231.7	224.0
SO_3	384.6	382.6	382.8	378.3	371.7
SeO_2	283.2	278.5	288.2	279.3	275.8
TeO_2	343.3	339.9	352.0	340.7	338.6
OCF_2	218.9	214.6	218.4	214.0	206.8
NOF	107.1	97.8	104.3	100.3	93.1
NO_2F	99.8	91.0	99.7	94.3	86.8
OPF_3	247.6	244.4	246.6	243.3	237.8
SOF_2	188.0	182.4	188.0	183.8	179.8
SOF_4	269.2	265.6	268.5	264.0	257.3
SO_2F_2	174.8	169.8	174.5	170.9	165.4
BeF_2	377.3	376.1	376.2	370.9	366.2
PF_3	204.8	200.5	204.2	201.1	196.5
PF_5	384.3	382.9	382.8	377.8	371.4
AsF_3	247.8	244.4	247.3	243.2	242.1
AsF_5	443.9	443.2	442.5	436.1	432.2
SbF_3	294.5	292.4	293.8	289.2	288.9
SbF_5	499.9	499.9	498.7	491.5	487.4
SF_2	205.6	199.7	205.6	201.5	196.6
SF_4	231.3	225.2	232.0	226.4	221.8
SeF_4	299.8	295.1	299.8	294.3	291.7
TeF_4	355.1	352.9	354.9	349.5	347.8
ClF_3	255.9	249.0	254.9	248.2	243.4
ClF_5	212.9	204.0	206.5	202.1	196.3
BrF_3	294.7	288.9	294.1	288.1	285.6
BrF_5	253.7	243.7	250.9	247.0	243.8
IF_3	323.1	319.5	323.5	318.5	317.0
IF_5	274.7	269.4	275.3	272.0	271.3
F_2CCO	273.7	267.4	271.8	266.4	258.4
BMe_3	248.8	248.3	248.2	244.4	242.1
BOH_3	184.8	184.2	184.5	181.7	178.5
$AlMe_3$	369.3	369.2	368.8	362.0	357.5
$AlOH_3$	378.9	378.4	378.4	372.4	371.0
Me_3C^+	829.6	833.6	829.8	826.1	822.8
Me_3Si^+	963.4	964.5	962.8	955.2	950.5

Table S9 continued.

LA	PBEh-3c	B97-3c	r²SCAN-3c	ωB97X-3c
F_2	179.1	207.7	198.7	119.7
HF	279.3	215.5	216.9	220.4
H_2O	183.7	138.6	138.6	138.6
H_2CCO	322.9	198.2	211.3	207.7
H_2CO	213.5	146.3	149.5	134.7
HCO_2H	202.3	121.5	129.1	124.9
HCN	320.1	227.9	241.4	238.9
BH_3	426.8	322.1	325.1	320.1
BF_3	494.9	364.4	377.3	393.6
BF_2Cl	532.6	398.6	412.0	418.1
$BFCl_2$	558.6	419.4	432.9	433.9
BCl_3	575.2	431.2	444.6	443.4
BBr_3	604.3	454.9	478.0	470.7
AlH_3	546.0	406.7	411.3	419.1
AlF_3	623.5	504.6	516.9	524.8
AlF_2Cl	646.6	518.8	529.2	530.5
$AlFCl_2$	666.3	529.1	537.5	534.1
$AlCl_3$	683.1	536.2	543.0	536.1
GaF_3	592.8	463.1	484.5	484.8
$GaCl_3$	613.3	460.5	479.2	462.9
InF_3	568.7	448.2	467.4	503.1
$InCl_3$	589.4	443.5	464.3	488.0
H_3C^+	1275.5	1153.7	1156.3	1129.8
F_3C^+	1283.3	1109.3	1128.4	1140.3
H_3Si^+	1273.0	1117.2	1140.9	1110.4
SiH_4	289.3	186.3	191.7	195.7
SiF_4	460.4	329.2	344.0	351.7
$SiCl_4$	494.9	356.6	368.3	350.0
F_3Si^+	1411.0	1256.2	1281.4	1290.7
GeF_4	485.6	371.8	390.2	396.1
$GeCl_4$	479.4	338.6	352.7	324.1
SnF_4	538.4	432.6	451.2	447.4
$SnCl_4$	525.8	386.1	404.7	379.6
BeO	747.4	596.8	621.3	611.9
CO	151.4	101.8	104.0	76.1
CO_2	291.4	171.4	182.6	191.2
SO_2	365.9	247.9	258.5	258.9
SO_3	517.9	358.5	377.3	406.9
SeO_2	418.2	289.9	303.2	312.7
TeO_2	471.0	334.6	355.2	386.2
OCF_2	352.3	239.7	255.7	258.0
NOF	180.8	161.6	162.1	125.6
NO_2F	193.0	153.3	153.9	136.1
OPF_3	403.9	271.2	279.9	294.5
SOF_2	315.8	234.7	238.1	235.0
SOF_4	422.0	303.5	306.5	321.8
SO_2F_2	334.4	221.8	225.6	224.1
BeF_2	523.0	383.2	404.4	422.2
PF_3	333.6	231.1	237.5	249.0
PF_5	534.4	403.8	410.7	435.9
AsF_3	363.7	272.7	282.4	291.0
AsF_5	567.4	446.5	464.4	485.2
SbF_3	413.3	321.0	334.2	337.5
SbF_5	623.6	504.4	528.2	539.7
SF_2	320.5	244.1	248.0	244.7
SF_4	364.9	286.0	286.3	286.6
SeF_4	421.5	337.9	345.2	356.8
TeF_4	481.3	387.1	400.1	428.2
ClF_3	357.8	336.7	323.8	297.4
ClF_5	319.0	388.3	348.3	273.9
BrF_3	401.0	356.0	354.5	350.5
BrF_5	365.9	378.0	359.0	348.0
IF_3	444.5	386.2	387.4	382.4
IF_5	364.9	286.0	286.3	360.4
F_2CCO	420.2	297.6	317.2	310.9
BMe_3	408.2	271.9	285.8	274.4
BOH_3	305.1	205.1	213.4	218.6
$AlMe_3$	531.3	382.6	396.0	392.6
$AlOH_3$	514.2	393.4	408.5	403.3
Me_3C^+	1004.0	838.0	858.3	850.1
Me_3Si^+	1138.8	963.6	991.5	973.2

Table S10: Mean absolute error (MAE), mean signed error (MSE), standard deviation (StD) and the maximum signed error (MaxE) in kJ/mol for direct FIA computations using the evaluated XC functionals, at CCSD(T*)-F12a/AVTZ-F12 structures. Except for the composite methods, def2-QZVPD basis sets have been used.

	SVWN	BP86-D4	B97-D	BLYP-D4
MAE	29.9	25.4	35.0	34.4
MSE	28.6	−11.4	−26.4	−21.1
StD	25.1	28.5	28.7	33.6
MaxE	95.1 (ClF_5)	92.0 (ClF_5)	83.9 (ClF_5)	111.8 (ClF_5)
	PBE-D4	TPSS-D4	r²SCAN-D4	M06-L-D4
MAE	24.3	17.9	11.9	11.4
MSE	−8.6	−3.4	9.6	−0.8
StD	28.5	23.1	15.7	16.0
MaxE	91.1 (ClF_5)	78.7 (ClF_5)	65.7 (F_2)	66.8 (F_2)
	MN15-L	PBE0-D4	TPSSh-D4	PW6B95-D4
MAE	10.3	8.7	11.9	6.7
MSE	−3.7	2.7	0.3	−0.4
StD	12.6	10.0	15.3	8.0
MaxE	−44.5 (BrF_5)	25.9 (H_3C^+)	47.8 (F_2)	−19.6 (Me_3C^+)
	M06-D4	MN15	M06-2X-D4	B3LYP-D4
MAE	8.3	9.9	16.9	15.5
MSE	−4.3	6.3	12.9	−8.8
StD	8.7	10.9	13.9	15.7
MaxE	−18.8 (F_3C^+)	−44.7 (ClF_5)	48.5 (TeO_2)	44.0 (ClF_5)
	BHLYP-D4	ωB97M-V	ωB97X	ωB97X-D
MAE	8.4	6.4	7.2	11.0
MSE	3.1	−3.2	−4.8	−9.9
StD	12.6	7.7	8.3	7.7
MaxE	−53.7 (ClF_5)	−25.7 (ClF_5)	−34.5 (ClF_5)	−23.1 (Me_3C^+)
	ωB97X-V	CAM-B3LYP-D4	LH07s-SVWN-D4	LH07t-SVWN-D4
MAE	6.8	6.8	17.2	12.8
MSE	−3.0	−1.9	−14.4	−8.9
StD	8.5	8.0	14.2	11.7
MaxE	−35.2 (ClF_5)	−21.8 (Me_3C^+)	−33.6 (Me_3Si^+)	27.7 (BrF_5)
	LH12ct-ssirPW92-D4	LH12ct-ssifPW92-D4	LH14t-calPBE-D4	LH20t-D4
MAE	12.6	13.1	10.3	5.8
MSE	−11.1	−11.6	−3.1	−0.1
StD	9.4	9.5	11.5	7.5
MaxE	−27.5 (SiF_4)	−28.8 (SiF_4)	30.9 (BrF_5)	25.5 (SeO_2)
	LHJ14	LHJ-HF	LHJ-HFcal-D4	mPSTS-noa2
MAE	14.0	7.5	7.8	16.1
MSE	9.4	−4.4	−4.0	−11.7
StD	16.2	8.3	9.1	14.6
MaxE	56.7 (ClF_5)	−23.3 (Me_3C^+)	−25.5 (ClF_5)	−35.3 (Me_3C^+)
	TMHF-D4	TMHF-3P	ωLH22t-D4	scLH22t-D4
MAE	12.5	13.5	5.3	6.2
MSE	−9.9	−12.3	−1.0	−0.4
StD	12.2	10.9	7.6	7.5
MaxE	−47.7 (Me_3C^+)	−46.9 (Me_3C^+)	25.1 (SeO_2)	20.8 (SeO_2)
	scLH22ta-D4	ωLH23tdE-D4	B2PLYP-D4	B2GP-PLYP-D4
MAE	16.8	5.4	12.4	7.0
MSE	−9.0	−1.3	−7.1	−4.0
StD	17.5	7.2	12.5	7.1
MaxE	58.4 (F_2)	−24.7 (ClF_5)	43.8 (ClF_5)	24.6 (ClF_5)
	B2K-PLYP	B2T-PLYP	PWPB95-D4	PBE0-DH-D3(BJ)
MAE	4.9	9.9	5.0	8.4
MSE	−3.4	−7.0	−1.1	7.2
StD	4.5	8.8	6.1	7.7
MaxE	12.7 (ClF_5)	26.2 (ClF_5)	21.8 (F_2)	29.6 (SeO_2)
	DSD-BLYP-D3(BJ)	DSD-PBEP86-D3(BJ)	DSD-PBEB95-D3(BJ)	Pr²SCAN50-D4
MAE	8.9	5.4	3.6	6.8
MSE	−5.7	−2.4	−2.7	6.7
StD	8.4	6.2	3.4	4.3
MaxE	31.5 (ClF_5)	25.3 (ClF_5)	11.2 (F_2)	25.9 (F_2)
	Pr²SCAN69-D4	ϰPr²SCAN50-D4	ωPr²SCAN50-D4	ωB97M(2)
MAE	3.7	5.8	3.2	4.4
MSE	3.5	5.8	0.8	−4.0
StD	2.5	3.9	4.1	3.0
MaxE	9.4 (H_3C^+)	17.8 (SeO_2)	13.2 (F_2)	−12.8 (F_3C^+)
	PBEh-3c	B97-3c	r²SCAN-3c	ωB97X-3c
MAE	142.0	34.9	44.3	43.2
MSE	142.0	34.3	44.3	43.2
StD	26.2	30.3	21.4	16.7
MaxE	189.2 (F_3C^+)	189.7 (ClF_5)	149.7 (ClF_5)	102.2 (BrF_5)

Table S11: Comparison of MAEs in kJ/mol obtained for the three different approaches for all methods, at CCSD(T*)-F12a/AVTZ-F12 structures. Except for the composite methods, def2-QZVPD basis sets have been used.

	SVWN	BP86-D4	B97-D	BLYP-D4
Direct	29.9	25.4	35.0	34.4
OCF_2	22.7	21.5	19.7	22.8
Me_3Si^+	40.1	35.7	27.5	39.8
	PBE-D4	TPSS-D4	r²SCAN-D4	M06-L-D4
Direct	24.3	17.9	11.9	11.4
OCF_2	22.1	16.8	11.7	18.0
Me_3Si^+	38.3	23.7	18.6	12.4
	MN15-L	PBE0-D4	TPSSh-D4	PW6B95-D4
Direct	10.3	8.7	11.9	6.7
OCF_2	9.4	9.0	12.0	6.7
Me_3Si^+	9.3	17.2	16.5	14.9
	M06-D4	MN15	M06-2X-D4	B3LYP-D4
Direct	8.3	9.9	16.9	15.5
OCF_2	7.7	7.5	8.4	11.9
Me_3Si^+	13.4	9.2	13.6	22.9
	BHLYP-D4	ωB97M-V	ωB97X	ωB97X-D
Direct	8.4	6.4	7.2	11.0
OCF_2	7.7	6.9	6.8	6.3
Me_3Si^+	7.6	14.4	15.0	11.1
	ωB97X-V	CAM-B3LYP-D4	LH07s-SVWN-D4	LH07t-SVWN-D4
Direct	6.8	6.8	17.2	12.8
OCF_2	8.7	7.4	12.1	9.7
Me_3Si^+	12.3	16.3	19.8	18.2
	LH12ct-ssirPW92-D4	LH12ct-ssifPW92-D4	LH14t-calPBE-D4	LH20t-D4
Direct	12.6	13.1	10.3	5.8
OCF_2	7.8	7.7	10.1	6.8
Me_3Si^+	15.6	15.4	18.8	12.5
	LHJ14	LHJ-HF	LHJ-HFcal-D4	mPSTS-noa2
Direct	14.0	7.5	7.8	16.1
OCF_2	13.9	7.2	8.3	11.7
Me_3Si^+	25.6	14.6	13.1	16.3
	TMHF-D4	TMHF-3P	ωLH22t-D4	scLH22t-D4
Direct	12.5	13.5	5.3	6.2
OCF_2	11.5	10.3	7.2	7.0
Me_3Si^+	22.8	21.6	12.8	14.4
	scLH22ta-D4	ωLH23tdE-D4	B2PLYP-D4	B2GP-PLYP-D4
Direct	16.8	5.4	12.4	7.0
OCF_2	13.2	7.2	8.0	4.7
Me_3Si^+	23.3	13.8	13.5	8.0
	B2K-PLYP	B2T-PLYP	PWPB95-D4	PBE0-DH-D3(BJ)
Direct	4.9	9.9	5.0	8.4
OCF_2	3.2	6.0	4.8	6.3
Me_3Si^+	5.7	11.1	8.8	9.5
	DSD-BLYP-D3(BJ)	DSD-PBEP86-D3(BJ)	DSD-PBEB95-D3(BJ)	Pr²SCAN50-D4
Direct	8.9	5.4	3.6	6.8
OCF_2	5.3	4.1	3.3	3.3
Me_3Si^+	9.5	8.0	6.3	4.1
	Pr²SCAN69-D4	κPr²SCAN50-D4	ωPr²SCAN50-D4	ωB97M(2)
Direct	3.7	5.8	3.2	4.4
OCF_2	2.1	3.3	3.6	2.9
Me_3Si^+	2.1	3.8	6.0	5.9
	PBEh-3c	B97-3c	r²SCAN-3c	ωB97X-3c
Direct	142.0	34.9	44.3	43.2
OCF_2	22.1	18.1	14.0	13.2
Me_3Si^+	38.0	32.5	16.3	31.1

Table S12: Directly calculated reaction energy ΔE^{FIA} in kJ/mol obtained with ωB97M-V and different basis sets (D_F indicates that diffuse functions are just used on fluorine atoms). At CCSD(T*)-F12a/AVTZ-F12 structures.

LA	def2-SVP	def2-SVPD$_F$	def2-SVPD	def2-TZVP	def2-TZVPP
F_2	195.3	93.6	93.6	121.0	121.0
HF	276.7	190.5	190.2	220.0	216.9
H_2O	198.0	102.4	116.8	142.7	141.0
H_2CCO	340.6	136.0	167.9	216.8	216.6
H_2CO	241.0	70.7	108.1	146.7	147.0
HCO_2H	240.9	55.1	95.3	136.7	137.2
HCN	343.0	171.5	193.5	248.7	250.8
BH_3	473.9	268.3	281.5	340.7	339.4
BF_3	524.0	352.6	352.6	395.6	395.6
BF_2Cl	555.0	359.1	381.8	424.1	424.1
$BFCl_2$	575.7	363.3	400.3	442.5	442.5
BCl_3	588.4	366.3	411.5	453.6	453.6
BBr_3	628.3	397.0	429.8	487.3	487.3
AlH_3	561.4	358.5	364.4	434.0	433.7
AlF_3	654.0	482.0	481.9	528.1	527.9
AlF_2Cl	665.9	480.2	489.8	538.1	537.9
$AlFCl_2$	674.5	479.5	495.8	545.3	545.1
$AlCl_3$	680.6	478.3	499.7	550.4	550.3
GaF_3	619.9	450.8	450.4	493.0	493.0
$GaCl_3$	615.8	410.0	436.8	482.3	482.3
InF_3	593.5	433.2	433.1	479.1	479.1
$InCl_3$	593.7	397.4	420.4	468.8	468.7
H_3C^+	1305.6	1090.9	1094.3	1154.7	1155.2
F_3C^+	1308.1	1091.1	1090.2	1145.3	1145.3
H_3Si^+	1274.0	1051.8	1050.3	1146.8	1146.5
SiH_4	322.4	137.7	149.4	223.9	223.5
SiF_4	504.3	343.4	342.8	358.1	357.9
$SiCl_4$	498.0	283.8	330.4	377.4	377.5
F_3Si^+	1435.1	1223.9	1225.7	1294.3	1294.1
GeF_4	533.3	368.8	368.0	404.1	404.1
$GeCl_4$	486.0	274.1	318.2	357.0	357.0
SnF_4	585.2	417.2	417.1	461.0	461.0
$SnCl_4$	529.1	329.1	361.2	405.7	405.6
BeO	746.6	581.0	590.1	618.3	618.3
CO	164.8	31.8	53.5	88.3	88.3
CO_2	310.9	112.1	151.1	193.1	193.1
SO_2	365.2	187.9	229.4	273.2	273.3
SO_3	529.4	335.2	360.3	431.4	431.3
SeO_2	420.8	237.1	280.4	330.4	330.4
TeO_2	474.3	287.4	331.0	394.6	394.6
OCF_2	385.9	206.2	221.8	264.3	264.3
NOF	204.1	91.8	97.9	130.6	130.6
NO_2F	233.4	86.2	99.1	136.8	136.8
OPF_3	437.8	267.1	277.0	297.6	297.7
SOF_2	336.6	201.2	207.7	229.9	229.8
SOF_4	472.1	295.5	299.6	319.6	319.5
SO_2F_2	363.4	200.5	205.4	225.8	225.6
BeF_2	553.2	373.7	372.1	414.0	414.0
PF_3	362.6	214.7	223.9	245.7	246.0
PF_5	602.9	416.1	415.3	431.6	431.9
AsF_3	398.6	254.7	258.3	288.8	288.8
AsF_5	632.3	449.3	448.1	488.3	488.3
SbF_3	450.1	300.2	302.1	336.3	336.3
SbF_5	685.4	500.7	500.7	544.1	544.1
SF_2	334.8	202.1	212.5	241.3	241.3
SF_4	397.6	249.7	252.7	274.1	274.2
SeF_4	462.6	312.6	314.9	344.3	344.3
TeF_4	527.9	367.6	369.2	399.3	399.3
ClF_3	384.6	252.9	255.8	279.9	279.7
ClF_5	367.1	236.3	232.8	226.1	225.3
BrF_3	427.0	296.0	299.1	326.6	326.6
BrF_5	406.9	276.9	275.4	286.8	286.8
IF_3	473.1	338.6	343.6	362.3	362.3
IF_5	450.6	315.3	314.0	324.3	324.3
F_2CCO	438.2	253.7	272.2	315.4	315.4
BMe_3	444.8	222.6	237.2	301.0	301.0
BOH_3	351.6	144.9	178.4	231.8	232.7
$AlMe_3$	549.4	334.4	345.0	412.9	412.5
$AlOH_3$	537.9	347.9	365.2	415.5	415.4
Me_3C^+	1030.5	811.3	812.1	876.6	876.1
Me_3Si^+	1142.8	916.4	917.6	1006.0	1005.7

Table S12 continued.

LA	def2-TZVPD$_F$	def2-TZVPD	def2-QZVP	def2-QZVPP	def2-QZVPD$_F$	def2-QZVPD
F_2	81.8	81.8	99.2	99.2	81.0	81.0
HF	186.6	186.6	200.1	200.1	184.2	184.2
H_2O	109.8	113.6	126.3	126.3	110.7	112.5
H_2CCO	156.3	163.0	188.0	188.0	162.8	164.4
H_2CO	93.9	103.3	123.0	123.0	100.6	103.4
HCO_2H	79.9	90.9	111.3	111.3	87.8	91.1
HCN	193.1	196.7	222.1	222.1	198.2	199.9
BH_3	281.5	283.0	306.9	306.9	282.6	283.4
BF_3	340.2	340.2	362.1	362.1	338.0	338.1
BF_2Cl	364.7	369.1	392.5	392.5	367.3	368.3
$BFCl_2$	380.7	387.5	412.0	412.0	386.2	387.6
BCl_3	390.0	398.8	423.9	423.9	397.7	399.5
BBr_3	422.9	427.9	453.1	453.0	426.9	428.9
AlH_3	373.6	376.0	404.1	404.1	378.9	379.5
AlF_3	474.3	474.3	500.1	500.1	476.0	476.0
AlF_2Cl	481.4	484.0	510.3	510.3	485.5	485.8
$AlFCl_2$	486.0	490.9	517.6	517.6	492.1	492.8
$AlCl_3$	488.5	495.6	522.7	522.7	496.7	497.7
GaF_3	441.2	441.2	464.0	463.9	440.9	440.9
$GaCl_3$	421.8	428.5	453.8	453.9	428.2	429.4
InF_3	425.2	425.3	448.9	448.9	425.3	425.3
$InCl_3$	407.8	412.5	438.6	438.6	413.0	413.8
H_3C^+	1092.4	1092.8	1119.5	1119.5	1093.7	1093.8
F_3C^+	1082.9	1083.1	1108.0	1108.0	1082.1	1082.1
H_3Si^+	1082.7	1082.4	1111.9	1111.9	1085.6	1085.7
SiH_4	166.5	169.5	195.2	195.2	170.9	172.2
SiF_4	305.7	305.7	327.6	327.6	303.9	303.9
$SiCl_4$	314.2	323.3	349.9	349.9	323.8	325.4
F_3Si^+	1231.2	1231.1	1259.5	1259.5	1233.2	1233.2
GeF_4	352.8	352.7	373.5	373.4	350.6	350.6
$GeCl_4$	295.8	303.5	329.3	329.5	303.4	305.0
SnF_4	405.9	405.8	429.7	429.7	406.2	406.2
$SnCl_4$	343.3	349.6	376.8	376.8	350.8	351.9
BeO	566.1	572.3	601.5	601.5	576.9	578.2
CO	45.8	50.8	66.5	66.5	48.0	50.8
CO_2	133.6	142.7	165.3	165.3	140.7	143.4
SO_2	222.3	230.8	251.8	251.8	228.6	232.6
SO_3	370.7	377.5	403.3	403.3	378.0	379.9
SeO_2	278.6	287.2	308.4	308.9	284.8	289.4
TeO_2	338.1	349.9	375.8	375.8	351.3	355.3
OCF_2	209.5	213.2	235.2	235.2	211.9	212.9
NOF	92.4	94.0	110.2	110.2	93.4	93.9
NO_2F	88.1	91.1	111.3	111.3	90.4	91.3
OPF_3	243.3	245.9	267.5	267.5	244.0	244.7
SOF_2	183.0	185.5	205.2	205.2	184.3	185.4
SOF_4	264.1	265.3	287.1	287.1	263.4	263.8
SO_2F_2	171.7	173.9	196.0	196.0	172.5	173.3
BeF_2	366.0	365.9	388.6	388.6	365.6	365.6
PF_3	200.2	202.6	221.6	221.6	201.4	202.5
PF_5	376.9	376.8	398.9	398.9	375.2	375.2
AsF_3	243.0	244.8	264.1	264.0	243.8	244.3
AsF_5	433.3	433.2	454.9	454.8	431.4	431.4
SbF_3	287.4	288.0	309.9	309.9	288.2	288.5
SbF_5	485.7	485.6	509.8	509.8	486.1	486.1
SF_2	196.2	199.5	218.9	218.9	198.4	199.8
SF_4	225.8	226.6	247.2	247.2	226.2	226.5
SeF_4	294.3	294.7	315.8	315.7	294.8	295.1
TeF_4	347.9	348.1	370.1	370.1	347.9	348.0
ClF_3	233.3	234.6	253.5	253.5	232.9	233.6
ClF_5	176.5	176.4	194.2	194.2	172.9	172.9
BrF_3	278.8	279.7	300.1	299.4	279.0	279.9
BrF_5	235.3	235.2	255.8	253.9	234.4	234.4
IF_3	312.6	314.1	334.6	334.6	312.9	313.4
IF_5	274.3	274.1	293.4	293.4	271.8	271.9
F_2CCO	259.5	263.8	286.1	286.1	263.0	264.6
BMe_3	237.4	239.8	265.6	265.6	239.3	239.8
BOH_3	170.8	179.3	201.3	201.3	175.9	178.1
$AlMe_3$	350.6	353.1	380.7	380.7	354.7	355.2
$AlOH_3$	355.9	365.7	390.8	390.8	365.5	367.7
Me_3C^+	813.0	812.7	839.4	839.4	813.1	813.1
Me_3Si^+	941.4	940.8	969.9	969.9	943.4	943.4

Table S13: MAE in kJ/mol obtained for the three different approaches with ωB97M-V and different basis sets (D_F indicates that diffuse functions are just used on fluorine atoms). At CCSD(T*)-F12a/AVTZ-F12 structures.

	def2-SVP		def2-SVPD_F	def2-SVPD
Direct	168.1		21.4	14.0
OCF_2	19.4		21.1	13.5
Me_3Si^+	22.1		37.3	49.7
	def2-TZVP	def2-TZVPP	def2-TZVPD_F	def2-TZVPD
Direct	47.7	47.6	7.7	6.5
OCF_2	6.5	6.6	6.8	7.1
Me_3Si^+	6.1	6.3	12.5	16.3
	def2-QZVP	def2-QZVPP	def2-QZVPD_F	def2-QZVPD
Direct	19.4	19.4	6.2	6.4
OCF_2	6.2	6.3	6.4	6.9
Me_3Si^+	10.7	10.7	13.9	14.4

Table S14: Directly calculated reaction energy ΔE^{FIA} in kJ/mol for BMe_3 and $B(C_6F_5)_3$ obtained at PNO-CCSD(T*)-F12a level using different numerical settings (PNO cutoffs). The calculations employed aug-cc-pVTZ-F12 basis sets on F^-, B and C atoms directly bound to boron, and aug-cc-pVDZ-F12 basis sets for all other atoms.

LA	domopt=normal	domopt=tight	reference[a]
BMe_3	245.9	246.3	247.4
$B(C_6F_5)_3$	442.0	442.0	---

[a]Canonical CCSD(T*)-F12a results, cf. Table S1.

Table S15: Comparison of reaction energies ΔE^{FIA} in kJ/mol with different approaches and basis sets for $B(C_6F_5)_3$ (PNO-CCSD(T*)-F12a reference value: 442.0 kJ/mol, see Table S14) using the ωB97M-V functional (VV10 included self-consistently). D_F indicates that diffuse functions are used only on all fluorine atoms, D_{F-} that diffuse functions are just used on the free and bound fluoride. The wall time of the calculation of $[FB(C_6F_5)_3]^-$ in minutes and the relative increase to the time with the corresponding def2-XZVP basis (X=T, Q) are also provided.

	def2-TZVP	def2-TZVPD$_{F-}$	def2-TZVPD$_F$	def2-TZVPD
FIA (direct)	498.0	432.9	438.1	437.9
FIA (anchored, OCF_2)	445.5	441.4	440.3	436.4
FIA (anchored, $SiMe_3^+$)	451.7	451.2	456.4	456.9
wall time[a]	65	65 (0 %)	80 (+23 %)	113 (+74 %)
wall time[a,b]	58	58 (0 %)	64 (+10 %)	77 (+33 %)
	def2-QZVP	def2-QZVPD$_{F-}$	def2-QZVPD$_F$	def2-QZVPD
FIA (direct)	462.6	436.1	437.5	437.5
FIA (anchored, OCF_2)	438.9	437.5	437.2	436.2
FIA (anchored, $SiMe_3^+$)	452.6	452.7	454.0	454.1
wall time[a,b]	188	192 (+2 %)	259 (+38 %)	437 (+132 %)
wall time[a,c]	114	115 (+1 %)	132 (+16 %)	202 (+77 %)

[a]Calculations were performed with a local version of Turbomole, based on release 7.8, using 16 cores on an AMD EPYC 7313 16-core processor. [b]Using analytical four-center integrals. [c]Using semi-numerical integration of the exact-exchange contribution ($senex, gridsize 1) and RI-J for the Coulomb integrals.

Table S16: ΔSCF electron affinity (EA) of the fluorine atom and ΔE^{FIA} of selected Lewis acids (in kJ/mol) calculated with the DSD-PBEP86-D3(BJ) and Pr^{2}SCAN69-D4 DHs using different correlation-consistent basis sets of aug-cc-pVXZ (X = D, T, Q, 5, 6) type,[1-6] and extrapolation to the complete basis set (CBS) limit. Comparison to def2-QZVPD basis-set results.[a]

	D	T	Q	5	6	CBS(T,Q,5)	CBS(Q,5,6)	def2-QZVPD
	DSD-PBEP86-D3(BJ)							
EA	320.6	322.9	326.2	327.5	328.4	329.5	329.6	323.6
BCl_3	387.1	400.0	399.8	398.2	397.9	395.5	397.8	397.6
F_2	136.3	121.9	121.2	120.1	119.9	118.8	119.6	119.2
ClF_5	304.5	247.5	236.2	225.2	222.8	210.3	221.4	223.9
H_3C^+	1076.9	1093.2	1094.7	1094.8	1094.7	1093.7	1094.8	1095.5
	Pr^{2}SCAN69-D4							
EA	310.7	311.8	315.0	316.4	317.3	318.5	318.7	312.2
BCl_3	397.1	409.3	409.3	407.7	407.3	404.9	407.2	407.2
F_2	128.8	113.3	112.5	111.4	111.1	110.0	110.8	110.7
ClF_5	286.4	227.5	216.2	205.3	202.7	190.2	201.3	204.0
H_3C^+	1085.9	1100.9	1102.4	1102.4	1102.4	1101.3	1102.4	1103.3

[a]CBS extrapolation used the formula $E_{DFT} = E_\infty + e^{-cX}$ for the DFT part and $E_{MP2} = E_\infty + b(X + c)^{-3}$ for the MP2 part[7,8] of the DH.

Table S17: FIA of BCl_3 calculated at CCSD(T) level with different extrapolations to the complete basis set (CBS) limit based on aug-cc-pVXZ (X=T,Q,5) basis sets. Comparison to the explicitly correlated CCSD(T*)-F12a/AVTZ-F12 calculations used for the FIA71 benchmark.

CCSD(T)/CBS(T,Q)	CCSD(T)/CBS(Q,5)	CCSD(T)/CBS(T,Q,5)	CCSD(T*)-F12a
405.7	401.0	398.5	398.9

Table S18: Comparison of electron affinities of the fluorine atom in kJ/mol from ΔSCF calculations and from the negative of the HOMO energy of the fluoride anion by Koopmans' theorem for all XC functionals of the present study using def2-QZVPD basis sets (except for the "3c" composite methods).

	EA (ΔSCF)	−E(HOMO)
SVWN	399.7	−113.5
BP86-D4	360.9	−120.2
B97-D	348.4	−132.4
BLYP-D4	356.9	−128.3
PBE-D4	355.0	−130.3
TPSS-D4	332.2	−132.9
r²SCAN-D4	319.7	−115.3
M06-L-D4	297.1	−141.1
MN15-L	310.7	−82.9
PBE0-D4	313.0	22.1
TPSSh-D4	317.6	−71.9
PW6B95-D4	326.1	41.7
M06-D4	319.7	22.8
MN15	321.7	113.8
M06-2X-D4	314.4	198.3
B3LYP-D4	331.9	−1.6
BHLYP-D4	280.0	192.0
ωB97M-V	324.1	275.0
ωB97X	326.6	277.6
ωB97X-D	323.1	217.8
ωB97X-V	325.3	282.4
CAM-B3LYP-D4	341.3	182.4
LH07s-SVWN-D4	348.6	23.7
LH07t-SVWN-D4	353.9	48.6
LH12ct-ssirPW92-D4	348.9	97.7
LH12ct-ssifPW92-D4	342.5	115.0
LH14t-calPBE-D4	343.7	53.6
LH20t-D4	326.9	110.3
LHJ14	378.3	17.5
LHJ-HF	351.8	118.3
LHJ-HFcal-D4	338.0	107.0
mPSTS-noa2	300.2	−68.4
TMHF-D4	360.2	82.2
TMHF-3P	357.8	97.3
ωLH22t-D4	332.4	274.2
scLH22t-D4	327.3	108.7
scLH22ta-D4	361.6	52.7
ωLH23tdE-D4	335.4	274.2
B2PLYP-D4	333.1	191.9
B2GP-PLYP-D4	327.4	269.6
B2K-PLYP	324.5	315.2
B2T-PLYP	327.6	237.9
PWPB95-D4	319.9	166.7
PBE0-DH-D3(BJ)	303.3	180.1
DSD-BLYP-D3(BJ)	329.8	290.5
DSD-PBEP86-D3(BJ)	323.6	295.6
DSD-PBEB95-D3(BJ)	315.8	263.7
Pr²SCAN50-D4	308.8	186.9
Pr²SCAN69-D4	312.2	304.6
ϰPr²SCAN50-D4	314.8	183.3
ωPr²SCAN50-D4	331.8	333.7
ωB97M(2)	329.2	273.9
PBEh-3c	123.1	−208.9
B97-3c	285.5	−305.1
r²SCAN-3c	269.9	−265.2
ωB97X-3c	287.5	143.8

Table S19: Reference value and directly calculated reaction energy ΔE^{FIA} in kJ/mol for OCF_2 obtained with a selection of XC functionals for different structures (CCSD(T*)-F12a/AVTZ-F12 vs. CCSD(T*)-F12a/AVTZ-F12).

LA	Ref.	BP86-D4	TPSS-D4	B3LYP-D4	ωB97M-V	LH20t-D4	DSD-PBEB95-D3(BJ)
OCF_2^{TZ}	211.7	197.0	203.9	200.2	212.9	214.7	206.5
OCF_2^{DZ}	211.7	197.5	204.4	200.6	213.0	214.9	206.6

Table S20: Uncorrected reaction energies and FIAs in kJ/mol at CCSD(T*)-F12a/AVTZ-F12//CCSD(T*)-F12a/AVTZ-F12 level obtained with scalar relativistic ECPs on the heavy atom and the ECP back-correction used for the reference data for compounds with forth row elements (see Computational Details in main text).

LA	ΔE^{FIA}	FIA	ECP correct.	LA	ΔE^{FIA}	FIA	ECP correct.
BBr_3	426.1	422.5	2.6	SeO_2	265.6	263.0	4.8
GaF_3	447.8	446.0	4.4	AsF_3	242.2	241.3	1.7
$GaCl_3$	432.2	429.8	5.9	AsF_5	431.0	429.0	5.7
GeF_4	349.1	347.6	4.5	SeF_4	297.2	296.7	−4.1
$GeCl_4$	297.7	295.4	7.5	BrF_3	299.4	298.7	−12.5
				BrF_5	279.6	281.7	−33.8

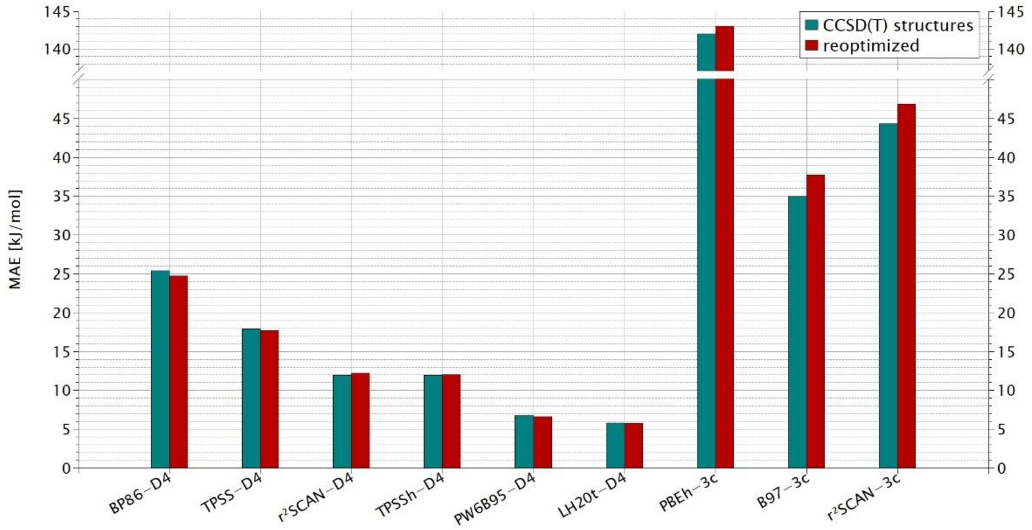

Figure S1: Summary of selected ΔE^{FIA} MAEs for direct computation with different functionals compared to the CCSD(T*)-F12a/AVTZ-F12//CCSD(T*)-F12a/AVTZ-F12 reference values, using either the CCSD(T*)-F12a/AVDZ-F12 reference structures or structures reoptimized with the given functional

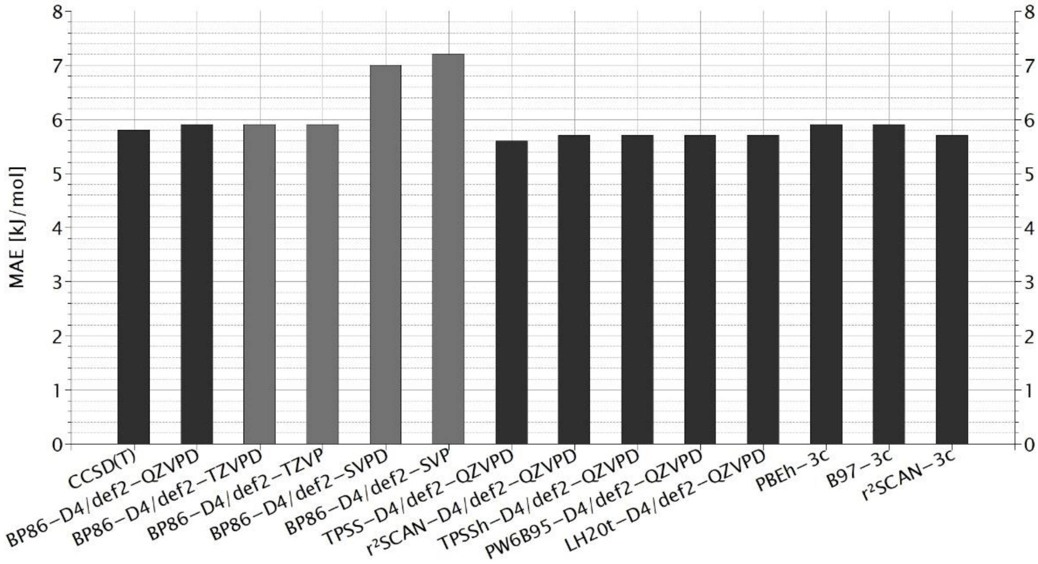

Figure S2: ΔE^{FIA} MAEs for direct computation at LH20t-D4/def2-QZVPD level compared to the CCSD(T*)-F12a/AVTZ-F12//CCSD(T*)-F12a/AVTZ-F12 reference values, using structures optimized at different levels.

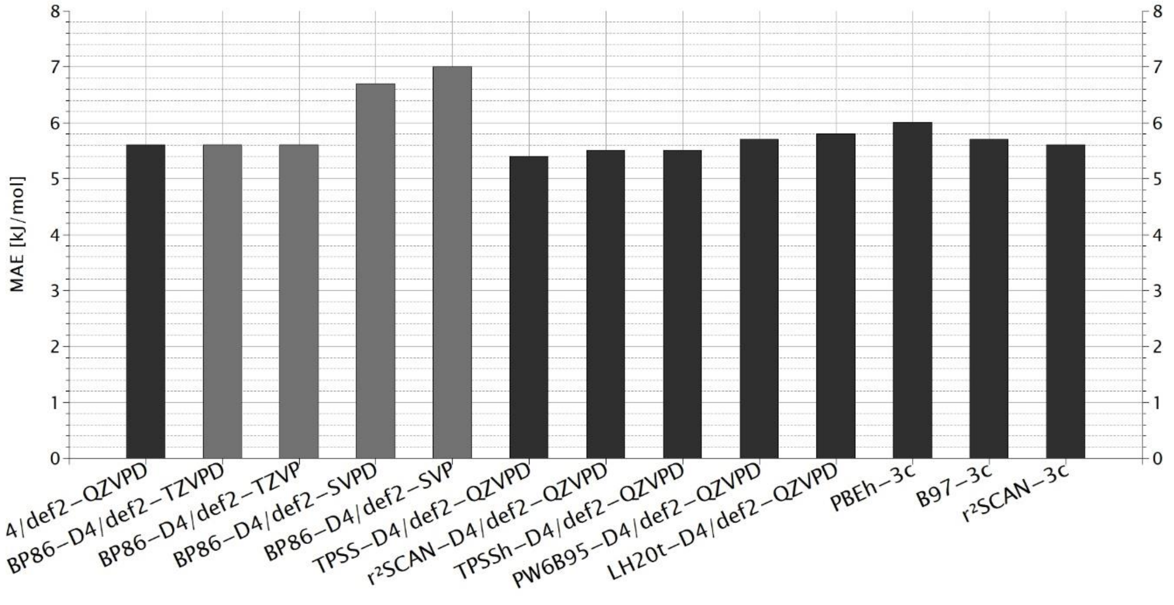

Figure S3: MAE of full FIAs for direct computation at LH20t-D4/def2-QZVPD level compared to the CCSD(T*)-F12a/AVTZ-F12//CCSD(T*)-F12a/AVTZ-F12 reference values, using structures optimized at different levels.

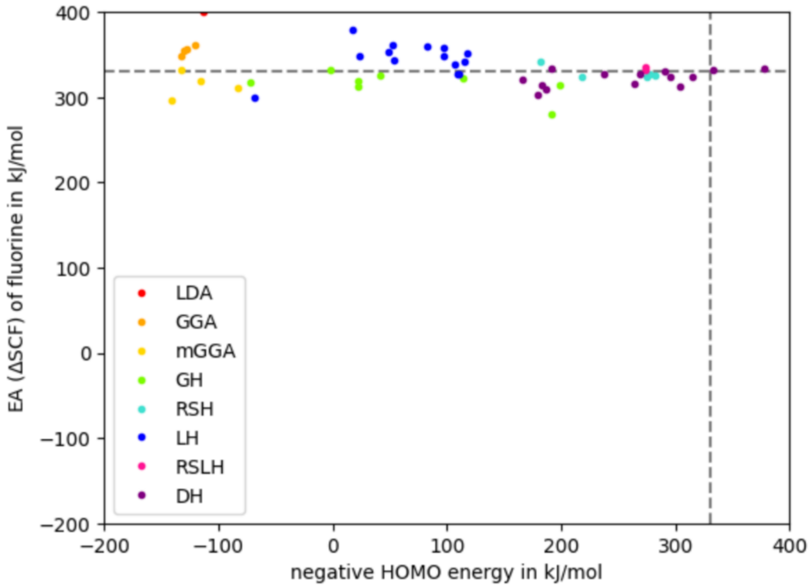

Figure S4: Comparison of ΔSCF results for the EA of the fluorine atom and the negative of the HOMO energy of the fluoride anion as Koopmans' theorem EA for different functionals (cf. Table S18).

References

1 T. H. Dunning Jr., *J. Chem. Phys.* **1989**, *90*, 1007–1023.

2 T. Van Mourik, T. H. Dunning Jr., *Int. J. Quantum Chem.* **2000**, *76*, 205–221.

3 T. Van Mourik, A. K. Wilson, T. H. Dunning JR, *Mol. Phys.* **1999**, *96*, 529–547.

4 A. K. Wilson, T. van Mourik, T. H. Dunning, *J. Mol. Struct.* **1996**, *388*, 339–349.

5 R. A. Kendall, T. H. Dunning Jr., R. J. Harrison, *J. Chem. Phys.* **1992**, *96*, 6796–6806.

6 D. E. Woon, T. H. Dunning Jr., *J. Chem. Phys.* **1993**, *98*, 1358–1371.

7 D. Feller, *J. Chem. Phys.* **1992**, *96*, 6104–6114.

8 T. Helgaker, W. Klopper, H. Koch, J. Noga, *J. Chem. Phys.* **1997**, *106*, 9639–9646.
